# Beta-Caryophyllene Prevents Ouabain-Induced Neurodegeneration and Behavioral Alterations Through PKA/GSK-3β Pathway

**DOI:** 10.1007/s11064-026-04843-2

**Published:** 2026-07-20

**Authors:** Jozyê Milena Silva Guerra, Adson Souza-Pereira, Luan Machado Maidana, Marizabel Parente Lins, Gabriel Lucca Martins Pereira, Mustafa Munir Mustafa Dahleh, Marina Prigol, Ana Flávia Furian, Mauro Schneider Oliveira, Leonardo Magno Rambo

**Affiliations:** 1 Biochemistry Graduate Program, Federal University of Pampa, Campus Uruguaiana, Uruguaiana, RS 97500-970 Brazil; 2 Biochemistry Graduate Program , Federal University of Pampa , Campus Itaqui, Itaqui, RS Brazil; 3 Physical Education Undergraduation, Federal University of Pampa, Campus Uruguaiana, Uruguaiana, RS Brazil; 4 Pharmacy Undergraduation, Federal University of Pampa, Campus Uruguaiana, Uruguaiana, RS Brazil; 5 Pharmacology Graduate Program, Federal University of Santa Maria, Santa Maria, RS Brazil

**Keywords:** Mood disorders, Neuroprotection, Antioxidant, Terpene, Redox Biomarkers

## Abstract

Bipolar disorder (BD) is a severe psychiatric condition characterized by recurrent mood episodes and progressive neurobiological alterations associated with oxidative stress, mitochondrial dysfunction, and neuronal damage. Current pharmacological treatments remain limited by incomplete efficacy and significant adverse effects, highlighting the need for novel therapeutic strategies. The present study investigated the neuroprotective effects of beta-caryophyllene (BCP), a natural sesquiterpene and selective cannabinoid receptor type 2 (CB2R) agonist, in a rat model of mania induced by intracerebroventricular ouabain (OUA) administration. Wistar rats received acute BCP treatment (three doses administered at 8-h intervals) starting one hour after OUA. Behavioral, biochemical, histological, and molecular analyses were performed seven days later. OUA induced manic-like behavioral alterations characterized by hyperactivity, increased risk-taking, and increased reactivity. These behavioral alterations were accompanied by increased lipid peroxidation, alterations in antioxidant enzyme activity, and enhanced neuronal degeneration in hippocampal regions, as indicated by Fluoro-Jade C staining. BCP treatment attenuated behavioral abnormalities, reduced oxidative damage, and prevented OUA-induced neuronal degeneration in the CA1, CA3, and dentate gyrus. Molecular analyses revealed that BCP restored phosphorylation of protein kinase A (PKA) and glycogen synthase kinase-3β (GSK-3β), while reversing the reduction of nuclear factor erythroid-2-related factor 2 (NRF2) expression induced by OUA. Together, these findings support the hypothesis that modulation of redox homeostasis and changes in PKA/GSK-3β/NRF2 signaling may contribute to the neuroprotective and behavioral effects of BCP. These findings provide preclinical evidence supporting further investigation of BCP and the molecular mechanisms that may underlie its effects in experimental models relevant to BD.

**Supplementary Information:**

The online version contains supplementary material available at 10.1007/s11064-026-04843-2.

## Introduction

Bipolar disorder (BD) is a prevalent psychiatric condition with a global prevalence of approximately 1–2% of the population and is characterized by mood fluctuations between manic and depressive episodes and is associated with functional and cognitive impairments [1]. The etiology of the disorder is multifactorial, involving genetic, neurochemical, and environmental factors, and its pathophysiology remains incompletely understood. Available evidence indicates that patients with BD exhibit elevated levels of oxidative stress markers, such as malondialdehyde (MDA), concomitant with reduced levels of endogenous antioxidants, including glutathione (GSH) [2]. In addition to redox alterations, structural neuroimaging studies have consistently reported volumetric reductions and cortical abnormalities in brain regions critically involved in mood regulation, including the hippocampus and prefrontal cortex, in individuals with BD [3, 4]. These structural changes are thought to reflect progressive neurobiological alterations associated with illness burden and episode recurrence. Accumulating evidence suggests that sustained oxidative stress and mitochondrial dysfunction may activate intrinsic apoptotic pathways, contributing to neuronal vulnerability and cell loss in BD [5–7]. Persistent redox imbalance may therefore not only disrupt synaptic function but also promote progressive neuronal damage in both hippocampal and cortical circuits, ultimately exacerbating mood dysregulation. A deeper comprehension of the roles of reactive oxygen species (ROS), oxidative stress, and antioxidant defenses in BD may prove instrumental in the development of innovative treatment strategies.

The treatment of BD remains a significant challenge, as adherence to therapy is often influenced by a multitude of individual, familial, social, and biological factors [8]. Indeed, the medications currently employed in the treatment of BD are associated with a range of adverse effects, which further complicate adherence to pharmacotherapy [9]. Given the limitations of conventional treatments for BD regarding tolerability, adherence, and even efficacy, current and future treatment challenges involve the development of medications and therapies that minimize adverse effects while improving treatment acceptability [10]. Furthermore, antioxidant therapies and those that modulate mitochondrial function represent promising research areas with the potential to improve clinical outcomes and quality of life for patients [11].

Bicyclic sesquiterpene beta-caryophyllene (BCP) is one of the most promising natural ligands of the cannabinoid receptor type 2 (CB2R). It is widely found in essential oils and in a variety of plants and dietary sources, including black pepper and clove [12]. BCP has attracted increasing attention in the context of neurological and psychiatric disorders due to its selective binding to CB2R and its well-documented anti-inflammatory, immunomodulatory, and antioxidant properties [13, 14]. Unlike CB1 receptor agonists, BCP does not produce psychoactive effects, which enhances its translational potential.

Growing evidence indicates that BCP exerts neuroprotective effects in several experimental models of central nervous system disorders. In models of neurodegenerative diseases, including Parkinson’s and Alzheimer’s diseases, BCP attenuated oxidative stress, reduced neuroinflammation, and preserved neuronal integrity through CB2R-dependent mechanisms [15, 16]. BCP has been shown to activate endogenous antioxidant defense systems, including the nuclear factor erythroid 2–related factor 2 (NRF2) signaling pathway, thereby enhancing cellular resilience against oxidative damage [17]. Beyond neurodegeneration, BCP has also demonstrated beneficial effects in models of mood-related disturbances. Experimental studies have shown that BCP produces anxiolytic- and antidepressant-like effects in rodents, which have been associated with modulation of monoaminergic neurotransmission, attenuation of neuroinflammatory processes, and reduction of oxidative stress markers [18–20]. In addition, CB2R signaling has been implicated in the regulation of dopaminergic pathways and neuroimmune responses relevant to mood disorders, suggesting that BCP may influence core neurobiological mechanisms involved in BD [21].

Although BCP has demonstrated neuroprotective, anxiolytic, antidepressant, and anti-inflammatory effects in several experimental models, including neurodegenerative and depressive-like conditions [13, 22, 23], its effects on manic-like behavior and neurobiological mechanisms associated with BD remain poorly investigated. Emerging evidence indicates that BCP may modulate intracellular signaling pathways involved in neuronal survival, including those associated with GSK-3β activity and apoptotic cascades [24]. These pathways are critically implicated in the pathophysiology of BD and overlap with mechanisms targeted by classical mood stabilizers such as lithium [25]. In this context, the OUA-induced model represents a well-established experimental approach that reproduces key behavioral and neurobiological features relevant to BD, including manic-like behavior, oxidative imbalance, neuroinflammation, and dysregulation of signaling pathways such as GSK-3β, PI3K/AKT/mTOR, and BDNF-related cascades [26–28]. However, the effects of BCP in this model, particularly its impact on the relationship between manic-like behavior, redox imbalance, and intracellular signaling, remain unexplored. Based on this evidence, we hypothesize that BCP exerts neuroprotective and behavioral effects in the OUA-induced model of mania by modulating intracellular signaling pathways involved in redox homeostasis and neuronal survival. In particular, these effects may involve modulation of the PKA/GSK-3β/NRF2 signaling axis, thereby contributing to antioxidant responses and neuroprotection. Based on this rationale, the present study aimed to evaluate the effects of acute BCP administration on behavioral and oxidative stress parameters during the manic-like phase in rats.

## Materials and Methods

### Animals and Reagents

Adult male Wistar rats (*n* = 24), 60 days old, were housed under controlled conditions (22 ± 2 °C, 45–55% humidity, 12 h light/dark cycle) with food and water *ad libitum*. The animals were obtained from the animal facility of the Federal University of Pampa (UNIPAMPA). All experimental procedures were conducted following the NIH guidelines for animal care and were approved by the institutional ethics committee (#007/2024). All efforts were made to reduce the number of animals used and minimize their suffering. All reagents were purchased from Sigma-Aldrich (USA), Santa Cruz Biotechnology (USA), MilliporeSigma (USA), Bio-Rad Antibodies (USA).

### Experimental Design

**Fig. 1:**
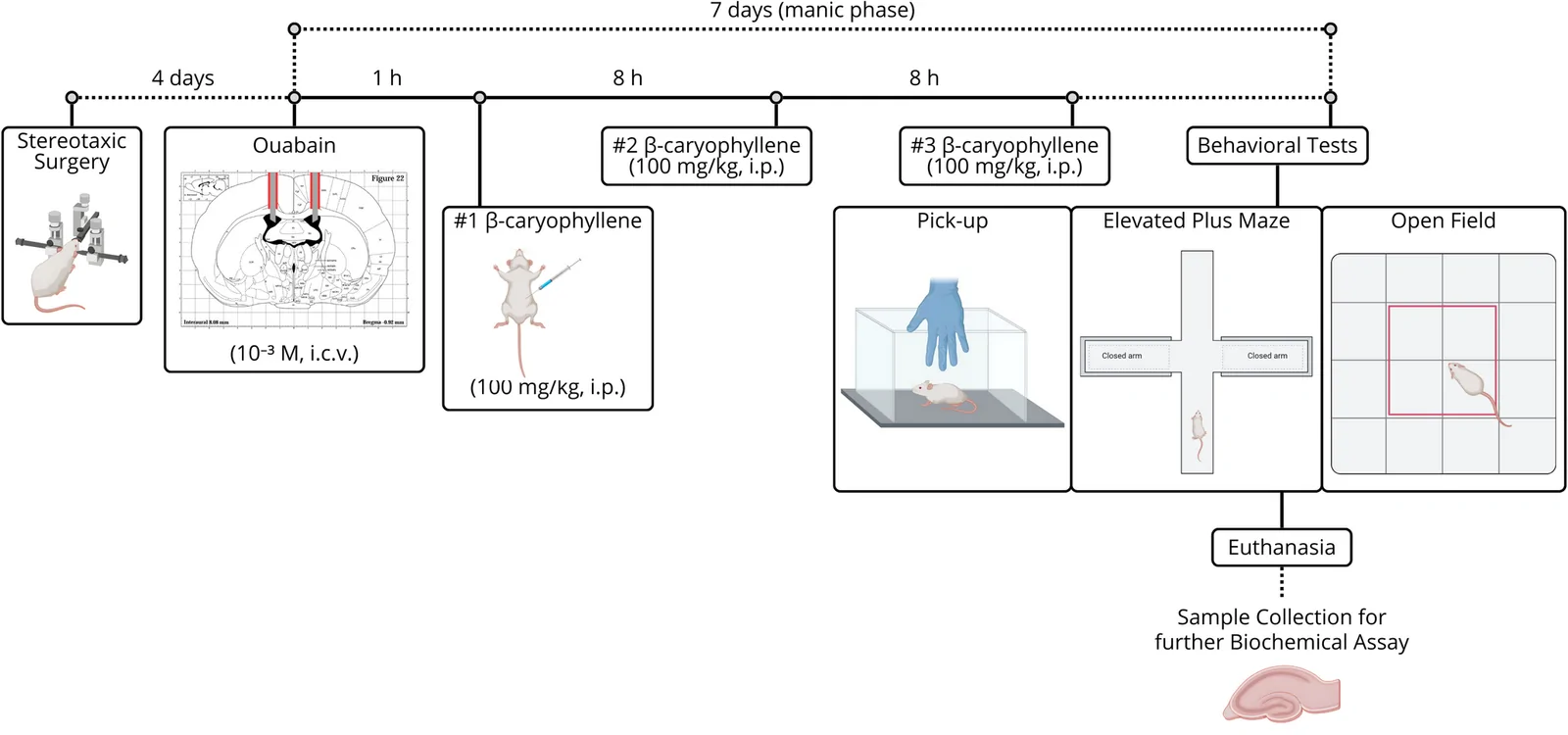
Timeline of the experimental design used to evaluate the effects of BCP on behavioral and biochemical changes in OUA-induced model of mania. After OUA injection (10^− 3^ M), BCP (100 mg/kg, i.p.) was administered at 1, 8, and 16 h after OUA injection. Behavioral tests occurred seven days later, followed by hippocampal collection for analysis

Animals were randomly divided into four experimental groups: Saline + aCSF; BCP + aCSF; Saline + OUA; BCP + OUA. All animals completed the full experimental protocol and were allocated to the experimental groups by simple alternation to ensure balanced group sizes. The experimental timeline is presented in Fig. 1. Following stereotaxic surgery, all animals received intracerebroventricular OUA injection (Saline + OUA and BCP + OUA groups) or vehicle (Saline + aCSF and BCP + aCSF groups) four days later. One hour post-injection, the BCP groups (BCP + aCSF and BCP + OUA groups) received their first BCP administration, followed by additional doses eight and sixteen hours later, the aforementioned methodology was replicated in the groups that received only the vehicle (Saline + aCSF and Saline + OUA groups). Behavioral tests were conducted seven days after OUA or vehicle administration, followed by euthanasia and hippocampal collection for neurochemical assays.

### Sample Size Determination

Sample sizes were determined based on pilot experiments and previously published studies using adult male Wistar rats and comparable experimental designs and outcome measures [26–28]. Prior data showed large treatment-related effects in both domains, including behavioral outcomes in the open-field test (mean difference = 63.7, Cohen’s d = 1.65) and immunoreactivity in western blot analyses (mean difference = 0.65, Cohen’s d = 2.62). Exploratory sample size calculations based on these effect sizes, assuming α = 0.05 and power = 0.80, supported the use of sample sizes within the range adopted in the present study. This approach balanced statistical power, expected experimental variability, and the ethical principle of minimizing animal use. All animals underwent behavioral testing and were subsequently perfused, with no exclusions at any time point. No pre-established inclusion or exclusion criteria were defined, and no animals died or were excluded during the experiments. Due to considerations regarding tissue availability, resource constraints, and prior experimental evidence indicating sufficient effect sizes, a subset of samples was used for specific analyses. Five animals were selected per group for histological analyses and six animals per group for biochemical assays and behavioral tests. Sample selection was executed by simple alternation process and supervised by an experimenter blinded to treatment allocation.

### Surgical Procedure

Animals were anesthetized with ketamine (75 mg/kg, i.p.), xylazine (10 mg/kg, i.p.), and acepromazine (2.5 mg/kg, i.p.) and placed in a rodent stereotaxic apparatus. The scalp was shaved, and the surgical area was disinfected with chlorhexidine (4%). A 2-cm midline incision was made in the scalp, and a 0.8-mm-diameter hole was drilled in the left parietal bone for the insertion of a 9 mm guide cannula, placed 1 mm above the right lateral ventricle according to the following coordinates from bregma (mm): AP −0.9, ML −1.5, and DV − 2.6. The cannula was fixed with dental acrylic cement and superglue, and the incision was sutured so that the upper extremity of the cannula remained exposed. Immediately after surgery, animals received dextrose (1 g/kg) plus dipyrone (200 mg/kg) in 1 mL of 0.9% saline (NaCl, i.p.). After surgery, the animals were monitored twice daily for 48 h by a licensed veterinarian. Monitoring included assessment of general condition (activity level, posture, and coat appearance), food and water intake, integrity of the surgical site, and specific signs of pain or discomfort. No additional doses of analgesics were required, as the animals showed no obvious signs of pain or discomfort during the observation period.

### Drug Administration

Four days after surgery, animals received a single injection of OUA (ouabain octahydrate; Sigma-Aldrich, St. Louis, MO, USA; Cat# O3125) (10^− 3^ M, 5 µL, i.c.v.) or vehicle (aCSF, 5 µL, i.c.v.). A Hamilton^®^ microsyringe connected to a polyethylene tube was used for the infusion, with the injection cannula extending 1.0 mm beyond the guide cannula to reach the left lateral ventricle [29]. Each infusion lasted four minutes to minimize solution efflux. BCP (β-Caryophyllene; Sigma-Aldrich, St. Louis, MO, USA; Cat# W225207) (100 mg/kg, i.p.) or vehicle (0.9% NaCl + 0.05% Tween 20, i.p.) was administered in three doses, starting one hour after OUA and repeated at 8 h and 16 h. OUA concentration and BCP dose were selected based on previous studies demonstrating the efficacy of these protocols in experimental models relevant to BD and other CNS disorders [19, 26, 28–30].

### Behavioral Analysis

#### Open Field

Rats were placed in a wooden arena (50 × 50 × 50 cm), and the floor was divided into sixteen equal squares. Each animal was gently placed in the center of the arena and allowed to explore freely for 5 min seven days after the OUA injection. The locomotor and exploratory activities were assessed based on the number of crossings, total distance traveled, number of visits to the central area, and time spent in the central area [31]. Sessions were video recorded using a fixed overhead camera, and behavioral parameters were quantified using ANY-maze software (Stoelting Co., Wood Dale, IL, USA; RRID: SCR_014289).

#### Elevated Plus Maze

The elevated plus-maze consisted of two open arms and two closed arms, each measuring 50 × 10 cm, with 40 cm high walls on the closed arms. The apparatus was raised 50 cm above the floor. During the test, animals were placed in the center and allowed to explore for five minutes. Measurements included time spent in open arms, and number of entries in the closed arms [32]. Sessions were video recorded (fixed overhead camera), and behavioral parameters were independently scored by three blinded evaluators; the mean value was used for statistical analysis.

#### Pick-Up Test

The pick-up test was used to assess handling-induced reactivity. Each animal was picked up around the torso, and its response was scored as (1) very easy, (2) easy with vocalizations, (3) some difficulty, the rat rears and faces the hand, (4) the rat freezes, with or without vocalizations, (5) difficult, the rat avoids the hand and moves away, (6) very difficult, rat behaves defensively and may attack [33]. Sessions were video recorded (fixed overhead camera), and responses were independently scored by three blinded evaluators; the mean value was used for statistical analysis.

### Biochemical Analysis

#### Sample Processing

After the experimental protocol ended, animals were anesthetized intraperitoneally (i.p.) with ketamine (100 mg/kg) plus xylazine (10 mg/kg), followed by transcardial perfusion with 600 mL of 0.9% NaCl solution containing heparin (5 IU/mL). The brain was immediately rinsed with cold aCSF and placed in a Petri dish on ice. The hippocampi were then separated, with the left hemisphere used for oxidative stress measurements and Western blot analyses and the right hemisphere reserved for histological analysis. Left hippocampal samples were immediately flash-frozen in liquid nitrogen and stored at − 80 °C until further processing. Subsequently, the tissue was homogenized in Tris-HCl buffer (50 mM, pH 7.4), centrifuged at 10,000xg for 10 min at 4 °C, and the supernatant (S1 fraction) was used to evaluate oxidative stress parameters. Protein content was determined using the colorimetric Bradford method, with bovine serum albumin (BSA; 1 mg/mL) as the standard [34]. Right hippocampal samples were processed as described below for Fluoro-jade C staining assay.

#### Lipid Peroxidation Levels

Lipid peroxidation was assessed by quantifying malondialdehyde (MDA) levels using the thiobarbituric acid reactive substances (TBARS) method [35]. Briefly, 200 µL of the sample was mixed with 500 µL of 0.8% TBA, 200 µL of 8.1% SDS, and 500 µL of acetic acid. The reaction mixture was incubated at 95 °C for 2 h. After cooling, the absorbance was measured at 532 nm. Results were expressed as nmol of MDA per mg of protein.

#### CAT Activity

CAT activity was assessed using Aebi’s method [36], monitoring the decomposition of H_2_O_2_ at 240 nm. The reaction mixture contained phosphate buffer (pH 7.0), H_2_O_2_ (0.3 mM), and the sample supernatant. Absorbance was recorded for 60 s at 25 °C. Results were expressed as U/mg of protein, where one unit (U) corresponds to the consumption of 1 µmol of H_2_O_2_ per minute.

#### Superoxide Dismutase Activity (SOD)

SOD activity was measured according to Misra and Fridovich [37], based on the enzyme’s ability to inhibit the oxidation of epinephrine by superoxide anions (O_2_⁻˙), resulting in H_2_O_2_. The reaction was initiated by adding 6–18 µL of the sample to a mixture containing 30 µL of 6 mM epinephrine and 260–242 µL of 57.7 mM sodium carbonate buffer (pH 10.2) at 30 °C. Absorbance was recorded at 480 nm every 10 s for 2 min. One unit of SOD was defined as the amount of enzyme required to inhibit epinephrine oxidation by 50%, with results expressed as U/mg protein.

#### Glutathione S-Transferase Activity (GST)

GST activity was assessed based on the conjugation of reduced glutathione (GSH) with 1-chloro-2,4-dinitrobenzene (CDNB) [38]. 50 µL of sample was mixed with 0.1 M potassium phosphate buffer (pH 7.5), 50 mM CDNB as the substrate, and 100 mM GSH to start the reaction. The absorbance was monitored at 340 nm at 30-second intervals over 2 min using a spectrophotometer. The activity was expressed as nmol of conjugated CDNB per minute per milligram of protein (nmol/min/mg protein).

#### Western Blotting

Western blot analysis was performed as previously described [39], with minor modifications. Hippocampal S1 fractions were diluted in radioimmunoprecipitation assay (RIPA) buffer and centrifuged at 12,700 rpm for 20 min at 4 °C. Supernatant was collected, and protein concentration was determined using the bicinchoninic acid (BCA) protein assay (Thermo Fisher Scientific), and all samples were adjusted to a final concentration of 2 µg/µL. Equal amounts of protein (30 µg per sample) were separated on a 9% SDS-polyacrylamide gel (SDS-PAGE) together with a prestained molecular weight marker (Precision Plus Protein™ Kaleidoscope™, Bio-Rad) and transferred to nitrocellulose membranes using the Trans-Blot^®^ Turbo™ Transfer System (Bio-Rad). Equal protein loading was confirmed with Ponceau S staining (Sigma-Aldrich, P7170). Following transfer, membranes were blocked with 5% BSA in TBS-T (Tris-buffered saline with 0.1% Tween-20) and washed twice for 10 min at room temperature. Membranes were incubated overnight at 4 °C with primary antibodies against NRF2, GSK-3β, phospho-GSK-3β (Ser9), PKA, and phospho-PKA (Ser96). After primary antibody incubation, membranes were washed twice for 10 min in TBS-T and incubated for 2 h at room temperature with anti-mouse or anti-rabbit horseradish peroxidase-conjugated secondary antibodies. Detailed information regarding all primary and secondary antibodies is provided in Supplementary Table S1. Protein bands were visualized using enhanced chemiluminescence (ECL) substrate (Pierce ECL, Bio-Rad), and signals were captured with the ChemiDoc XRS+ Imaging System (Bio-Rad). Band intensities were quantified using Image Lab software (Bio-Rad, Hercules, CA, USA; RRID: SCR_014210) and normalized to total protein using Ponceau S staining [40]. Data are expressed as a percentage of the control group.

#### Fluoro-Jade C (FJC) Staining

The right hippocampal hemisphere was post-fixed in 4% paraformaldehyde (PFA) for 24 to 48 h. After immersion in 30% sucrose for three days, the tissues were rapidly frozen in liquid nitrogen and stored at − 80 °C. Coronal sections (16 μm thick) were obtained using a cryostat (CM2850, Lupetec), mounted on gelatin-coated slides, and left to dry at room temperature for 2 h.

For FJC staining, slides were sequentially immersed in 0.05% sodium hydroxide in 80% ethanol for 5 min, 70% ethanol for 2 min, distilled water for 2 min, and 0.06% potassium permanganate for 10 min. Sections were then incubated for 1 h in a solution containing 0.01% FJC (#2578699, MilliporeSigma) and 0.01% DAPI (#sc-3598, Santa Cruz Biotechnology) in 0.1% acetic acid. After staining, slides were rinsed with distilled water, cleared in xylene for 1 min, mounted with DPX, coverslipped, and left to dry at room temperature.

Images from the cerebral cortex and hippocampus were acquired using a 20× objective lens on a Zeiss AX10 ScopemA1 microscope equipped with a Zeiss Axiocam 105 color camera. Image acquisition was performed with Zen software (version 2.3, Zeiss), and analysis was conducted using Fiji software (version 1.51 J; RRID: SCR_002285). The “Color Threshold” function was used to isolate and enhance the specific labeling. FJC-positive cells were counted manually. For each animal, three sections containing the region of interest were analyzed. Quantitative measurements obtained from these sections were averaged, and the resulting mean value was used for statistical analysis.

### Statistical Analyses

Data were expressed as mean and ± SEM, or median (interquartile range). The Shapiro-Wilk test was used to assess the normality, and the data analysis was conducted using two-way analysis of variance (ANOVA) or Scheirer–Ray–Hare test, for parametric or non-parametric data, respectively. Post-hoc analyses were performed using the Tukey test; Correlation analyses were carried out using Pearson’s correlation coefficient. *p* < 0.05 was considered significant. Data analysis was performed using GraphPad Prism 8.0 (GraphPad Software, San Diego, CA, USA; RRID: SCR_002798). To improve data transparency, descriptive statistics for all outcome variables, including group means ± SD or medians and interquartile ranges, are provided in Supplementary Table S4.

## Results

**Fig. 2:**
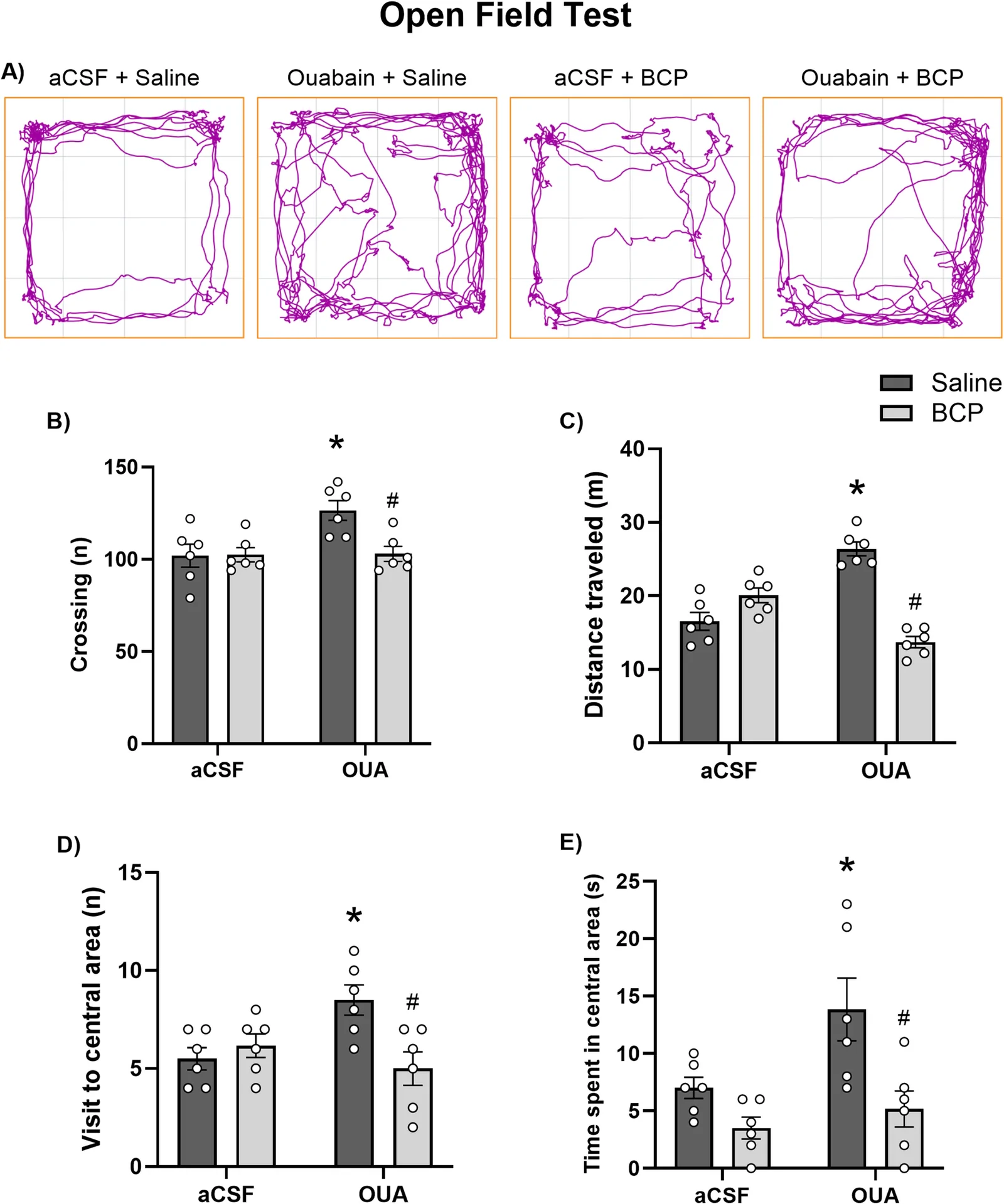
Effect of BCP treatment on locomotor activity and exploratory behavior in OUA-infused animals. **A** Representative open-field tracking plots showing locomotor paths during a 5-min test session in animals from the aCSF + Saline, OUA + Saline, aCSF + BCP, and OUA + BCP groups. **B**–**E** Quantification of the number of crossings **B**, total distance traveled **C**, number of visits to the central area **D**, and time spent in the central area **E**. Data are expressed as mean ± standard error of the mean (SEM); *n* = 6 animals per group. *p < 0.05 vs. aCSF + Saline; #*p* < 0.05 vs. OUA + Saline (two-way ANOVA followed by Tukey’s post hoc test)

We first evaluated the effects of BCP treatment on OUA-induced behavioral alterations in the open-field test, elevated plus maze, and pick-up test. In this sense, we found that BCP treatment reduced OUA-induced increase in number of crossings [F(1, 20) = 5.92; *p* = 0.015] (Fig. 2B), distance traveled [F(1, 20) = 67.40; *p* < 0.001] (Fig. 2C), visits to the central area [F(1, 20) = 8.71; *p* = 0.011] (Fig. 2D) and time spent in central area [F(1, 20) = 12.57; *p* = 0.001] (Fig. 2E) in the open field.

**Fig. 3:**
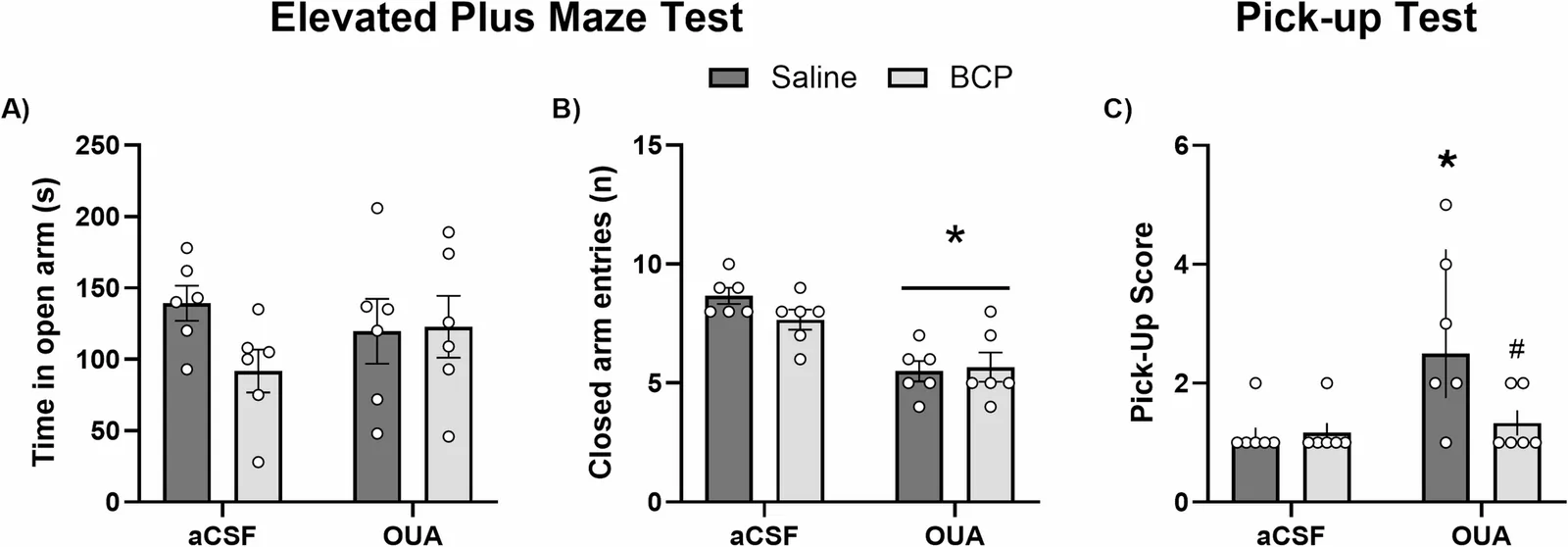
Behavioral parameters evaluated in the elevated plus maze (EPM) and pick-up test. **A** Time spent in the open arms of the EPM, **B** number of closed arm entries, and **C** pick-up score reflecting reactivity-related behavior. Data are expressed as mean ± standard error of the mean (SEM) (**A**, **B**) and as median with interquartile range **C**; *n* = 6 animals per group. * above the line indicates main effect; *p < 0.05 vs. aCSF + Saline; #*p* < 0.05 vs. OUA + Saline (two-way ANOVA followed by Tuckey’s post hoc test)

In the elevated plus maze, OUA administration decreased the number of entries into the closed arms of the apparatus [F(1, 20) = 31.41; *p* < 0.001; main effect] (Fig. 3B), without affecting the time spent in the open arms [F(1, 20) = 0.09; *p* = 0.761] (Fig. 3 A). To assess handling-induced reactivity animals were subjected to a pick-up test. The statistical analysis revealed that BCP treatment reduced the OUA-induced increase in pick-up score [F(1, 20) = 8.11; *p* = 0.046] (Fig. 3 C).

**Fig. 4:**
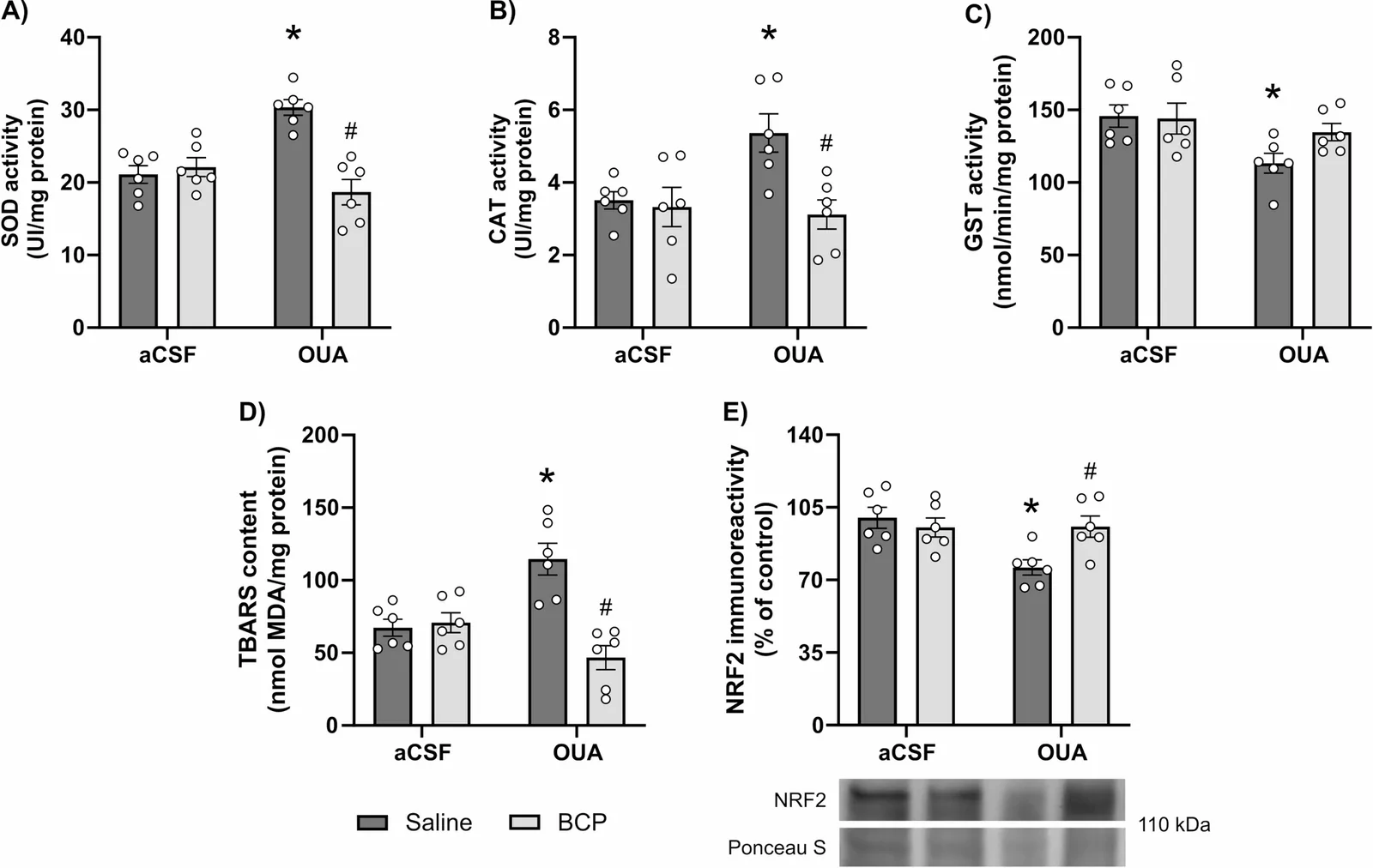
Effect of BCP on oxidative stress markers and antioxidant defenses in hippocampus of OUA-infused animals. **A**–**C** Enzymatic activities of superoxide dismutase (SOD; **A**), catalase (CAT; **B**), and glutathione S-transferase (GST; **C**). **D** Levels of lipid peroxidation, assessed by TBARS content. **E** Representative Western blot and quantification of NRF2 immunoreactivity normalized to Ponceau S staining. Data are expressed as mean ± standard error of the mean (SEM); *n* = 6 animals per group. *p < 0.05 vs. aCSF + Saline; #*p* < 0.05 vs. OUA + Saline (two-way ANOVA followed by Tuckey’s post hoc test)

Oxidative stress markers and antioxidant defenses were evaluated in hippocampal homogenates and are expressed in Fig. 4. Statistical analysis revealed that BCP treatment decreased OUA-induced increase in SOD [F(1, 20) = 21.57; *p* < 0.001] (Fig. 4A) and CAT activities [F(1, 20) = 5.41; *p* < 0.002] (Fig. 4B) as well as partially blunted OUA-induced decrease in GST activity [F(1, 20) = 6.96; *p* = 0.041] (Fig. 4C). In addition, we found that BCP treatment reversed the OUA-induced increase in TBARS content [F(1, 20) = 18.92; *p* < 0.001] (Fig. 4D).

**Fig. 5:**
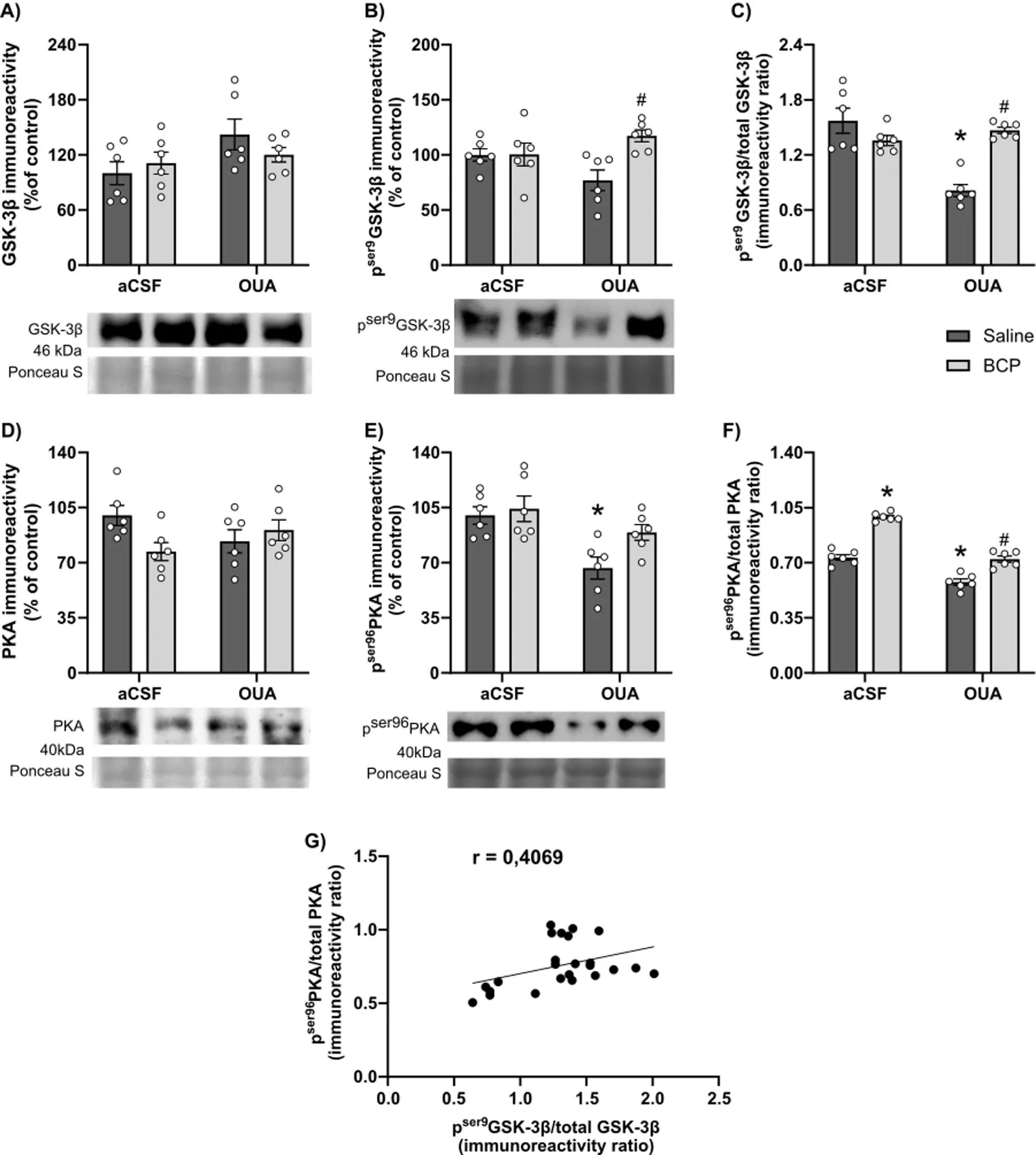
Effect of BCP on GSK-3β and PKA signaling in hippocampus of OUA-infused animals. **A**–**C** Representative Western blot images and quantification of total GSK-3β (**A**), phosphorylated GSK-3β at serine 9 (p^Ser9^GSK-3β; **B**), and the p^Ser9^GSK-3β/total GSK-3β ratio (C). **D**–**F** Representative Western blot images and quantification of total PKA (**D**), phosphorylated PKA at serine 96 (p^Ser96^PKA; **E**), and the p^Ser96^PKA/total PKA ratio (**F**). Pearson’s correlation analyses between p^Ser96^PKA/total PKA ratio and p^Ser9^GSK-3β/total GSK-3β ratio (**G**). Data are expressed as mean ± standard error of the mean (SEM); *n* = 6 animals per group. *p < 0.05 vs. aCSF + Saline; #*p* < 0.05 vs. OUA + Saline (two-way ANOVA followed by Tukey’s post hoc test)

To further investigate the molecular mechanisms underlying the behavioral and neurochemical effects of BCP in the OUA model of mania, we assessed hippocampal levels of GSK-3β, PKA, and NRF2 by Western blotting. No significant differences were observed among treatments for total GSK-3β immunoreactivity [F(1, 20) = 1.70; *p* = 0.206] (Fig. 5 A). For the phosphorylated (Ser9) GSK-3β, we found an increase in the interaction group (OUA + BCP) [F(1, 20) = 6.28; *p* = 0.021] (Fig. 5B). In addition, analysis of the phospho/total GSK-3β ratio revealed that BCP treatment reversed the OUA-induced decrease in GSK-3β phosphorylation [F(1, 20) = 27.83; *p* < 0.001] (Fig. 5C), suggesting that BCP restored GSK-3β phosphorylation, a modification associated with reduced kinase activity. No alterations were found in PKA total protein immunoreactivity among groups [F(1, 20) = 1.47; *p* = 0.234] (Fig. 5D). OUA administration significantly decreased phosphorylated PKA (Ser96) immunoreactivity [F(1, 20) = 13.86; *p* = 0.001] (Fig. 5E). Nevertheless, analysis of the phosphorylated/total PKA ratio demonstrated that BCP treatment effectively reversed the OUA-induced decrease in PKA phosphorylation [F(1, 20) = 10.12; *p* = 0.005] (Fig. 5F). Finally, assessment of NRF2 expression revealed that BCP treatment reversed the OUA-induced reduction of NRF2 immunoreactivity [F(1, 20) = 6.92; *p* = 0.016] (Fig. 4E). Together, these findings are consistent with the involvement of PKA, GSK-3β, and NRF2 signaling in the effects of BCP. Pearson’s correlation analysis revealed that the phosphorylated/total PKA ratio positively correlates with phospho/total GSK-3β ratio (*r* = 0.4069; *p* = 0.049) (Fig. 5G).

**Fig. 6:**
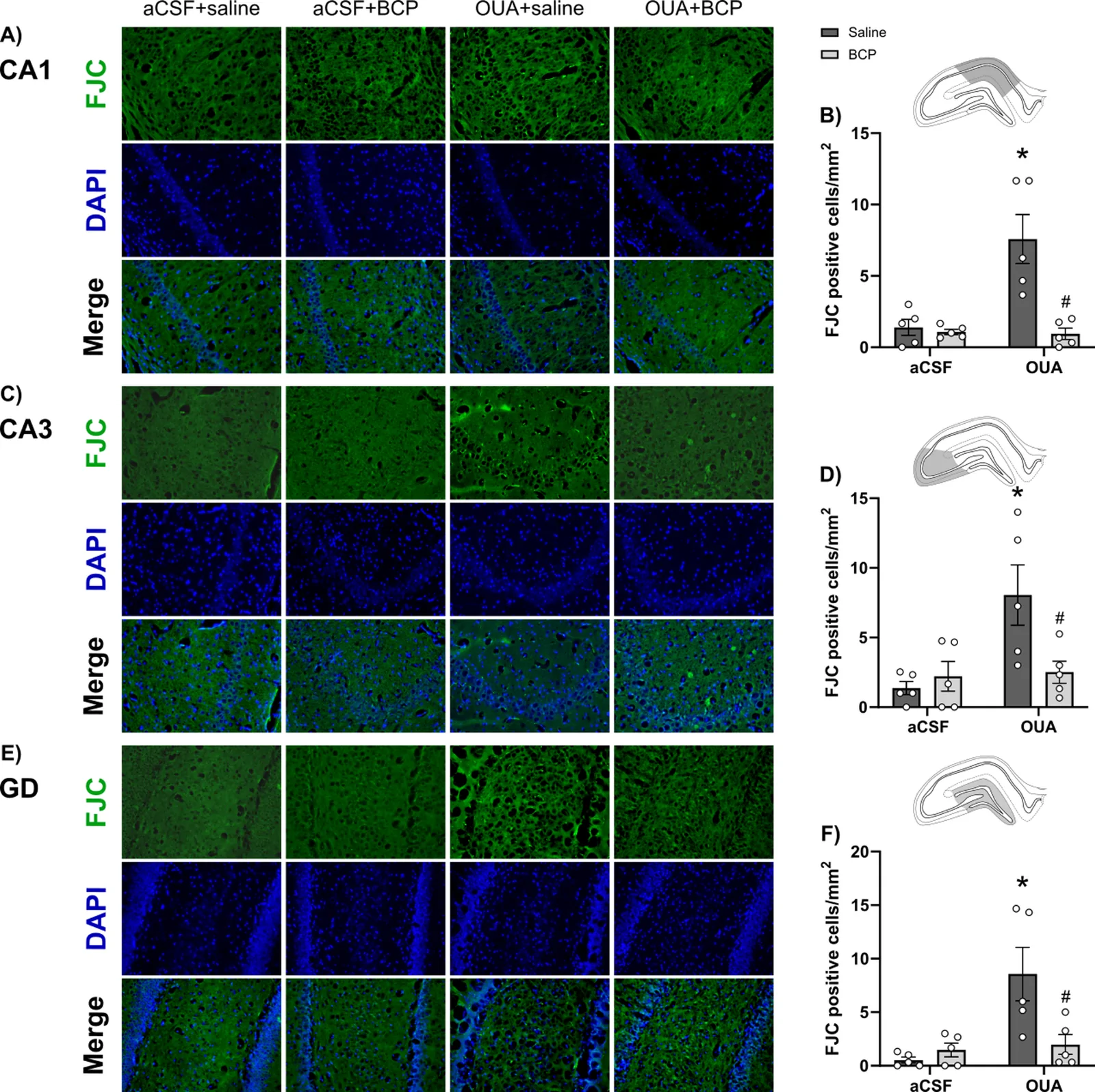
BCP reduces OUA-induced neurodegeneration in hippocampal subfields. **A**–**F** Representative photomicrographs and quantification of FJC-positive cells in the hippocampal CA1 (**A**, **B**), CA3 (**C**, **D**), and dentate gyrus (DG; **E**, **F**) regions. FJC staining (green) identifies degenerating neurons, and nuclei were counterstained with DAPI (blue). Insets show the corresponding hippocampal regions analyzed. Data are expressed as mean ± standard error of the mean (SEM); *n* = 5 animals per group. *p < 0.05 vs. aCSF + Saline; #*p* < 0.05 vs. OUA + Saline (two-way ANOVA followed by Tukey’s post hoc test). Scale bar = 50 μm

To evaluate the effect of BCP treatment on OUA-induced neuronal degeneration, FJC staining was performed in hippocampal sections encompassing the CA1, CA3, and dentate gyrus (DG) regions. Statistical analysis revealed that BCP treatment effectively prevented the OUA-induced increase in FJC labeling in all regions analyzed: CA1 [F(1, 16) = 11.59; *p* = 0.004] (Fig. 6B), CA3 [F(1, 16) = 6.11, *p* = 0.025] (Fig. 6D), and DG [F(1, 16) = 7.53; *p* = 0.014 (Fig. 6 F)].

Representative photomicrographs illustrate that OUA markedly increased the number of FJC-positive cells and the number of degenerating neurons in the CA1, CA3, and DG subfields, while animals treated with BCP exhibited staining patterns similar to control groups. These results demonstrate that BCP exerts a neuroprotective effect against OUA-induced neuronal damage in the hippocampus.

**Fig. 7:**
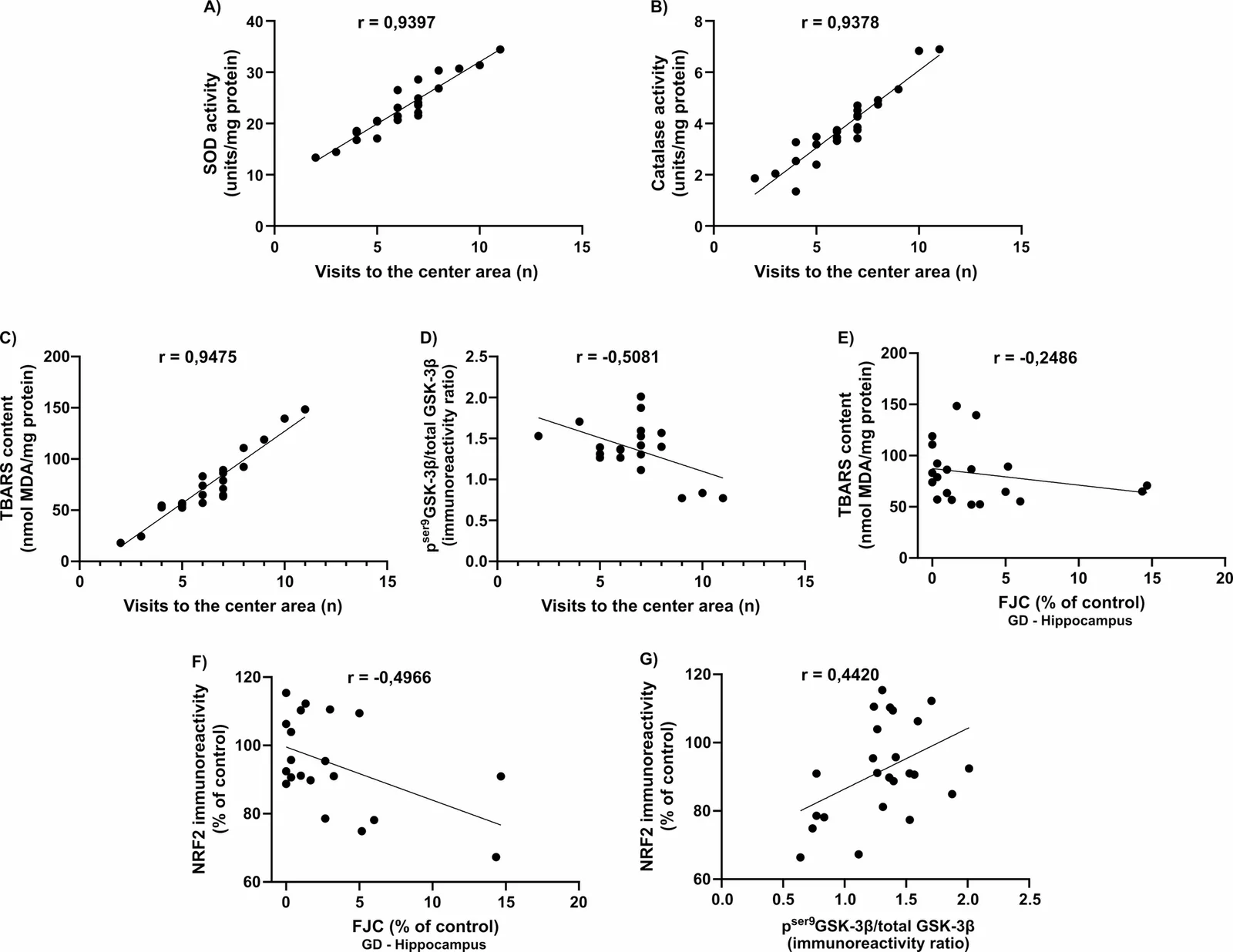
Pearson’s correlation analyses between (**A**) superoxide dismutase (SOD) activity, **B** catalase (CAT) activity, **C** thiobarbituric acid reactive substances (TBARS) content in rat hippocampal homogenates, and (**D**) p^Ser9^GSK-3β/total GSK-3β immunoreactivity ratio with the number of visits to the center zone in the Open Field (OF) test. TBARS content (**E**) and NRF2 immunoreactivity (**F**) were correlated with FJC staining (% of control) in the hippocampal dentate gyrus (DG), and NRF2 immunoreactivity (**G**) was correlated with the p^Ser9^GSK-3β/total GSK-3β immunoreactivity ratio. Each dot represents one animal; solid lines indicate least-squares linear regression. Pearson’s r values are shown in each panel.

Correlation analysis (Pearson’s) disclosed that the number of visits to the central area in the open field test seven days after OUA injection positively correlates with SOD (*r* = 0.9397; *p* < 0.001) (Fig. 7A), CAT (*r* = 0.9378; *p* < 0.001) (Fig. 7B), TBARS (*r* = 0.9475; *p* < 0.001) (Fig. 7C) and p^Ser9^GSK-3β/total GSK-3β immunoreactivity ratio (*r*= −0.5081; *p* = 0.022) (Fig. 7D). FJC-positive cells in the hippocampal dentate gyrus (DG) region was negatively correlated with NRF2 immunoreactivity (*r*= −0.4966; *p* = 0.026) (Fig. 7F) but was not significantly correlated with TBARS levels (*r*= −0.2486; *p* = 0.291) (Fig. 7E). In addition, NRF2 immunoreactivity positively correlates with p^Ser9^GSK-3β/total GSK-3β immunoreactivity ratio (*r* = 0.4420; *p* = 0.031) (Fig. 7G).

## Discussion

Previous studies have demonstrated neuroprotective and antidepressant-like effects of BCP in models of neurodegeneration and mood-related disorders; however, its effects on manic-like states and BD-associated neurobiological alterations remain largely unexplored. In this study, we demonstrated that acute BCP administration attenuated behavioral and neurochemical alterations induced by OUA. Specifically, BCP treatment, administered one hour after OUA injection and followed by two additional doses at eight-hour intervals, reduced behavioral features associated with the manic-like phenotype and mitigated oxidative stress and antioxidant enzyme alterations seven days after OUA administration. These findings are consistent with the hypothesis that modulation of oxidative stress and intracellular signaling pathways associated with neuronal survival and mood regulation may contribute to the observed effects of BCP.

Signaling processes in the brain are influenced by cellular and mitochondrial metabolism, as well as the equilibrium between the production and neutralization of ROS [41]. In this context, mitochondrial dysfunction and oxidative stress are linked to various neurological and psychiatric disorders [42]. Individuals with BD exhibit increased levels of ROS, resulting in oxidative stress that damages cellular components such as lipids, proteins, and DNA [6]. Persistent oxidative damage can impair mitochondrial function, activate intrinsic apoptotic pathways, and ultimately lead to neuronal cell death [43]. Oxidative damage and neuronal cell death impair synaptic plasticity, potentially worsening mood episodes and cognitive deficits [44]. In line with this rationale, our FJC findings demonstrated that OUA increased neuronal degeneration in the hippocampal subfields CA1, CA3, and dentate gyrus, whereas BCP treatment markedly attenuated this effect. Since FJC selectively labels degenerating neurons, these results suggest that OUA-induced oxidative imbalance is accompanied by structural neuronal damage, which was prevented by BCP administration. Our biochemical findings further support this interpretation. BCP treatment attenuated OUA-induced increases in MDA content and modulated antioxidant enzyme activity, indicating improved redox homeostasis during the manic-like state. The restoration of NRF2 expression together with normalization of the phospho/total GSK-3β and PKA ratios is consistent with the hypothesis that modulation of this signaling axis may contribute to the neuroprotective profile of BCP. NRF2 is a key transcriptional regulator of cellular antioxidant defenses, controlling the expression of multiple genes involved in ROS detoxification and redox homeostasis. The restoration of NRF2 levels by BCP may therefore contribute to the regulation of endogenous antioxidant systems and protection against oxidative damage. Significant correlations between oxidative parameters and open field behavior are consistent with the hypothesis that redox dysregulation may contribute to manic-like behaviors and that the behavioral improvement observed after BCP treatment may be associated with reduced ROS production and preservation of neuronal integrity.

Consistent with this interpretation, the alterations observed in antioxidant enzyme activity further suggest that OUA induces a robust oxidative challenge in the brain. In the present study, OUA increased SOD and CAT activity, whereas BCP treatment normalized these enzymatic changes. This increase in antioxidant enzyme activity likely represents a compensatory response to excessive ROS generation triggered by OUA. NRF2 regulates the transcription of several antioxidant enzymes, including SOD and CAT, which constitute major components of the endogenous defense system against oxidative stress. Therefore, the restoration of NRF2 expression observed after BCP treatment may contribute to the re-establishment of coordinated antioxidant responses in the brain. The relationship between SOD and CAT is closely intertwined in the ROS catabolism, with the product of SOD acting as the primary substrate for CAT, ultimately yielding neutral products [45]. Disruption to the activity of either enzyme can lead to an overload of the other, resulting in ROS accumulation and heightened vulnerability to oxidative stress [46]. Thus, the normalization of SOD and CAT activity by BCP may reflect a reduction in ROS burden and restoration of redox balance rather than suppression of antioxidant defenses. The combined action of SOD and CAT is of paramount importance in conditions where oxidative stress is a prominent feature, such as inflammation, neurological and neuropsychiatric disorders, including BD [47].

Beyond its capacity to attenuate oxidative stress by reducing ROS production and modulating antioxidant enzyme activity, BCP demonstrated remarkable efficacy in reducing risk-taking behavior and handling-induced reactivity during the manic-like phase. These findings indicate that BCP modulates neurochemical alterations while also attenuating behavioral changes associated with the manic-like phenotype induced by OUA. However, further studies are needed to determine whether these represent independent therapeutic effects or are part of the same mechanistic cascade. Risk-taking and aggressive behaviors are hallmark features of manic episodes in BD, often leading to detrimental personal, social, and financial outcomes. Despite the availability of mood-stabilizing agents such as lithium and valproate, these treatments usually fail to adequately address the impulsivity and aggression associated with mania [48, 49]. Therefore, identifying compounds like BCP that can simultaneously regulate neurochemical markers and behavioral manifestations of mania could contribute to the identification of novel therapeutic targets for BD.

BCP effects on risk-taking and aggressive/responsive behavior may be linked to its action on the endocannabinoid system, particularly its agonistic activity at the CB2 receptor. This receptor is known to exert anti-inflammatory and neuroprotective effects [50], which are essential for restoring neuronal homeostasis during pathological states like mania [51]. Evidence suggests that endocannabinoid system modulation can influence dopaminergic and serotonergic pathways [52], both of which are implicated in the regulation of impulsivity and aggression [53]. These mechanisms represent plausible pathways through which BCP may influence the behavioral alterations observed in the present study.

BCP treatment increased PKA phosphorylation, suggesting that modulation of this kinase may contribute to its neuroprotective effects. In the present study, we assessed PKA signaling by measuring phosphorylation at Ser96 in the regulatory subunit IIα (RIIα) [54]. This residue undergoes autophosphorylation mediated by the catalytic subunit and is therefore dependent on prior enzymatic activity of PKA. Although this site does not represent a canonical marker of PKA activation, its phosphorylation reflects the functional state and dynamic regulation of the PKA holoenzyme. Thus, the observed changes are interpreted as modulation of PKA signaling rather than direct evidence of its activation. This interpretation is consistent with the parallel changes observed in GSK-3β phosphorylation, a well-established PKA target.

Although CB2 receptors are classically coupled to Gi proteins and negatively regulate adenylate cyclase activity, accumulating evidence indicates that CB2R activation can indirectly modulate cAMP/PKA signaling through redox-sensitive mechanisms and interactions with dopaminergic pathways, particularly under conditions of oxidative stress and neuroinflammation [50, 52]. CB2R signaling is not restricted to a uniform canonical pathway and may vary according to the cellular and pathological context [55, 56]. In addition to receptor subtype, factors such as cell type, brain region, and disease state can influence the recruitment of distinct intracellular signaling cascades. In our study, these contextual factors include the hippocampal environment and the oxidative stress condition induced by OUA administration. Consistent with this interpretation, evidence from an experimental model of ischemic stroke suggests that BCP may engage cAMP/PKA signaling pathways in hippocampal tissue. Chen and colleagues reported that BCP increased hippocampal cAMP levels and PKA phosphorylation, whereas pharmacological inhibition of PKA attenuated its neuroprotective effects [57]. Although obtained in a different experimental context, these findings support the biological plausibility of the increased PKA phosphorylation observed in the present study.

It is important to consider that the effects of BCP are unlikely to be mediated exclusively by the signaling pathways investigated in the present study. Although our findings are consistent with the hypothesis that the PKA/GSK-3β/NRF2 axis may contribute to the effects of BCP, the experimental design does not allow definitive conclusions regarding the causal contribution of CB2R activation or the sequential involvement of these signaling components. This signaling axis should be considered a plausible mechanistic hypothesis that warrants further investigation rather than a definitively established mechanism. In addition to its role as a selective CB2R agonist, BCP has been reported to exert antioxidant, anti-inflammatory, and mitochondrial protective effects in experimental models of neurological injury. These actions include attenuation of neuroinflammatory responses, reduction of oxidative stress, and preservation of mitochondrial function, all of which may contribute to neuronal survival and behavioral improvement [22, 58, 59]. Therefore, mechanisms beyond the PKA/GSK-3β/NRF2 pathway may have contributed to the neuroprotective and behavioral effects observed in the present study. Further studies using pharmacological or genetic approaches will be necessary to determine the relative contribution of these pathways.

We observed parallel increases in PKA phosphorylation and GSK-3β phosphorylation at Ser9, a well-established inhibitory site [60]. GSK-3β is a critical kinase implicated in mood regulation and BD pathophysiology, and its inhibition represents a central mechanism of action of classical mood stabilizers such as lithium [61, 62]. The observed increase in Ser9 phosphorylation is consistent with reduced GSK-3β activity and raises the possibility that modulation of PKA signaling may be involved in this response, potentially contributing to the neuroprotective effects observed after BCP treatment.

GSK-3β has been shown to negatively regulate NRF2 by promoting its nuclear export and proteasomal degradation via β-TrCP–dependent mechanisms [63–65]. Thus, inhibition of GSK-3β favors NRF2 stabilization and transcriptional activation of antioxidant response element (ARE)-driven genes. OUA reduced NRF2 immunoreactivity, whereas BCP treatment restored NRF2 levels, suggesting a potential association between GSK-3β inhibition and NRF2 regulation in this experimental context. Although previous studies have demonstrated that GSK-3β can regulate NRF2 stability, the present findings do not establish a causal relationship between these molecular changes, which should therefore be interpreted as being consistent with, rather than demonstrating, the proposed signaling mechanism. The parallel normalization of NRF2 expression and oxidative stress markers further supports this interpretation but does not allow causal inference.

The molecular changes induced by BCP were paralleled by reduced FJC staining in hippocampal subfields, consistent with attenuation of neuronal degeneration. Since excessive GSK-3β activity has been associated with apoptotic signaling, mitochondrial dysfunction, and increased neuronal vulnerability [66], its inhibition by BCP may have contributed to the attenuation of OUA-induced neurodegenerative processes. Activation of NRF2 promotes cellular resistance to oxidative damage and limits neuronal death [67]. These results suggest that signaling pathways involving PKA, GSK-3β, and NRF2 may contribute to the neuroprotective and behavioral effects of BCP.

Another notable advantage of BCP is its safety profile. As a natural sesquiterpene found in various dietary sources, BCP is associated with low toxicity and high tolerability [68], supporting its continued evaluation in preclinical and translational studies. Its potential relevance to BD warrants further investigation, particularly in combination with existing mood stabilizers, to assess potential synergistic effects on neurochemical and behavioral outcomes. Future research should aim to elucidate the precise neurobiological mechanisms through which BCP modulates behavior, with a particular focus on its impact on the endocannabinoid system and related signaling pathways. In addition, expanding investigations to female animals will be important to determine whether the effects observed here are consistent across sexes, thereby strengthening the translational relevance of these findings. Clinical studies are also necessary to evaluate its efficacy and safety in human populations, as well as to determine optimal dosing strategies for behavioral and neurochemical modulation in BD.

In conclusion, BCP attenuated behavioral and neurobiological alterations induced by OUA and was associated with molecular changes consistent with modulation of oxidative stress-related pathways. Its ability to attenuate risk-taking behavior, handling-induced reactivity, oxidative damage, and neuronal degeneration highlights its neuroprotective potential in this experimental model. These findings provide preclinical evidence supporting further investigation of BCP as a potential therapeutic strategy in experimental models of BD.

## Supplementary Information

Below is the link to the electronic supplementary material.

Supplementary Material 1

## Data Availability

The datasets generated and/or analyzed during the current study are available from the corresponding author upon reasonable request.

## Abbreviations

aCSF: Artificial cerebrospinal fluid
ANOVA: Analysis of variance
ARE: Antioxidant response element
BCA: Bicinchoninic acid
BCP: β-Caryophyllene
BD: Bipolar disorder
BDNF: Brain-derived neurotrophic factor
BSA: Bovine serum albumin
CAT: Catalase
CB2R: Cannabinoid receptor type 2
CDNB: 1-Chloro-2,4-dinitrobenzene
CNS: Central nervous system
DG: Dentate gyrus
ECL: Enhanced chemiluminescence
EPM: Elevated plus maze
FJC: Fluoro-jade C
GSH: Reduced glutathione
GST: Glutathione S-transferase
MDA: Malondialdehyde
NRF2: Nuclear factor erythroid 2-related factor 2
OUA: Ouabain
PFA: Paraformaldehyde
PI3K: Phosphoinositide 3-kinase
PKA: Protein kinase A
ROS: Reactive oxygen species
RIPA: Radioimmunoprecipitation assay
SDS: Sodium dodecyl sulfate
SDS-PAGE: Sodium dodecyl sulfate polyacrylamide gel electrophoresis
SEM: Standard error of the mean
SOD: Superoxide dismutase
TBARS: Thiobarbituric acid reactive substances
TBA: Thiobarbituric acid
TBS-T: Tris-buffered saline with tween-20
