# Supplementary material for: Beta-Caryophyllene Prevents Ouabain-Induced Neurodegeneration and Behavioral Alterations Through PKA/GSK-3β Pathway
Source: Neurochem Res. 2026 Jul 20;51(4):222. doi: 10.1007/s11064-026-04843-2 (PMC13384979; doi:10.1007/s11064-026-04843-2)
Supplement: Supplementary file 1 — Supplementary Material 1 [file 11064_2026_4843_MOESM1_ESM.docx]

**Supplementary data**

| **Table S1. Primary and secondary antibodies used in Western Blotting assays** | | | | | | |
| --- | --- | --- | --- | --- | --- | --- |
| **Antibody** | **Manufacturer** | **Catalog Number** | **Host Specie** | **Dilution** | **RRID** | **Supporting Reference** |
| NRF2 | Bio-Rad | VMA00224 | Mouse | 1:1000 | AB_3752226 | Ghosh et al., 2020; Lau et al., 2013 |
| GSK-3β | Sigma-Aldrich | G7914 | Mouse | 1:1000 | AB_477027 | Ma et al., 2012 |
| p-GSK-3β (Ser9) | Sigma-Aldrich | ZRB1557 | Rabbit | 1:1000 | AB_3752227 | Souza-Pereira et al., 2025 |
| PKA | Santa Cruz Biotechnology | sc-365615 | Mouse | 1:1000 | AB_10846720 | Manufacturer datasheet |
| p-PKA (Ser96) | Santa Cruz Biotechnology | sc-56941 | Mouse | 1:1000 | AB_2168241 | Manufacturer datasheet |
| Goat anti-mouse IgG-HRP | Santa Cruz Biotechnology | sc-2005 | Goat | 1:10000 | AB_631736 | Manufacturer datasheet |
| Goat anti-rabbit IgG-HRP | Sigma-Aldrich | A6154 | Goat | 1:10000 | AB_258284 | Manufacturer datasheet |

| **Table S2. Two-way ANOVA results** | | | | |
| --- | --- | --- | --- | --- |
| **Variable** | **Factor** | **F value** | **p value** | **effect size (η²)** |
| Crossing | interaction | F (1, 20) = 5.920 | 0.0245 | 0.228 |
|  | ouabain | F (1, 20) = 6.423 | 0.0197 | 0.243 |
|  | β-caryophyllene | F (1, 20) = 5.437 | 0.0303 | 0.214 |
| Time spent in central area | interaction | F (1, 20) = 2.268 | 0.1477 | 0.102 |
|  | ouabain | F (1, 20) = 6.137 | 0.0223 | 0.235 |
|  | β-caryophyllene | F (1, 20) = 12.57 | 0.0020 | 0.386 |
| Distance traveled | interaction | F (1, 20) = 67.40 | 0.0001 | 0.771 |
|  | ouabain | F (1, 20) = 3.138 | 0.0917 | 0.136 |
|  | β-caryophyllene | F (1, 20) = 21.32 | 0.0002 | 0.516 |
| Visit to central area | interaction | F (1, 20) = 8.705 | 0.0079 | 0.303 |
|  | ouabain | F (1, 20) = 1.685 | 0.2090 | 0.078 |
|  | β-caryophyllene | F (1, 20) = 4.025 | 0.0585 | 0.168 |
| Time in open arms | interaction | F (1, 20) = 1.894 | 0.1839 | 0.087 |
|  | ouabain | F (1, 20) = 0.095 | 0.7614 | 0.005 |
|  | β-caryophyllene | F (1, 20) = 1.450 | 0.2425 | 0.068 |
| Closed arm entries | interaction | F (1, 20) = 1.601 | 0.2203 | 0.074 |
|  | ouabain | F (1, 20) = 31.41 | 0.0001 | 0.611 |
|  | β-caryophyllene | F (1, 20) = 0.817 | 0.3768 | 0.039 |
| Pick-up score | interaction | F (1, 20) = 4.044 | 0.0580 | 0.168 |
|  | ouabain | F (1, 20) = 8.115 | 0.0099 | 0.289 |
|  | β-caryophyllene | F (1, 20) = 4.044 | 0.0580 | 0.168 |
| GST | interaction | F (1, 20) = 2.110 | 0.1619 | 0.095 |
|  | ouabain | F (1, 20) = 6.957 | 0.0158 | 0.258 |
|  | β-caryophyllene | F (1, 20) = 1.519 | 0.2320 | 0.071 |
| SOD | interaction | F (1, 20) = 21.57 | 0.0002 | 0.519 |
|  | ouabain | F (1, 20) = 4.520 | 0.0462 | 0.184 |
|  | β-caryophyllene | F (1, 20) = 15.29 | 0.0009 | 0.433 |
| CAT | interaction | F (1, 20) = 5.410 | 0.0306 | 0.213 |
|  | ouabain | F (1, 20) = 3.475 | 0.0771 | 0.148 |
|  | β-caryophyllene | F (1, 20) = 7.531 | 0.0125 | 0.274 |
| TBARs | interaction | F (1, 20) = 18.92 | 0.0003 | 0.486 |
|  | ouabain | F (1, 20) = 2.011 | 0.1716 | 0.091 |
|  | β-caryophyllene | F (1, 20) = 15.36 | 0.0008 | 0.434 |
| NRF2 | interaction | F (1, 20) = 6.919 | 0.0160 | 0.257 |
|  | ouabain | F (1, 20) = 6.461 | 0.0194 | 0.244 |
|  | β-caryophyllene | F (1, 20) = 2.626 | 0.1208 | 0.116 |
| total GSK-3β | interaction | F (1, 20) = 1.704 | 0.2065 | 0.079 |
|  | ouabain | F (1, 20) = 4.098 | 0.0565 | 0.170 |
|  | β-caryophyllene | F (1, 20) = 0.185 | 0.6721 | 0.009 |
| phospho GSK-3β | interaction | F (1, 20) = 6.276 | 0.0210 | 0.239 |
|  | ouabain | F (1, 20) = 0.159 | 0.6991 | 0.008 |
|  | β-caryophyllene | F (1, 20) = 6.576 | 0.0185 | 0.247 |
|  |  |  |  |  |
|  |  |  |  |  |
| **Table S2. Two-way ANOVA results (continued)** | | | | |
| **Variable** | **Factor** | **F value** | **p value** | **effect size (η²)** |
| GSK-3β ratio | interaction | F (1, 20) = 27.83 | 0.0001 | 0.582 |
|  | ouabain | F (1, 20) = 15.46 | 0.0008 | 0.436 |
|  | β-caryophyllene | F (1, 20) = 7.130 | 0.0147 | 0.263 |
| total PKA | interaction | F (1, 20) = 5.259 | 0.0328 | 0.208 |
|  | ouabain | F (1, 20) = 0.046 | 0.8317 | 0.002 |
|  | β-caryophyllene | F (1, 20) = 1.471 | 0.2393 | 0.069 |
| phospho PKA | interaction | F (1, 20) = 1.992 | 0.1735 | 0.091 |
|  | ouabain | F (1, 20) = 13.86 | 0.0013 | 0.409 |
|  | β-caryophyllene | F (1, 20) = 4.235 | 0.0529 | 0.175 |
| PKA ratio | interaction | F (1, 20) = 10.12 | 0.0047 | 0.336 |
|  | ouabain | F (1, 20) = 142.8 | 0.0001 | 0.877 |
|  | β-caryophyllene | F (1, 20) = 129.9 | 0.0001 | 0.867 |
| CA1 Fluoro-Jade C | interaction | F (1, 16) = 11.59 | 0.0036 | 0.420 |
|  | ouabain | F (1, 16) = 10.63 | 0.0049 | 0.399 |
|  | β-caryophyllene | F (1, 16) = 14.03 | 0.0018 | 0.467 |
| CA3 Fluoro-Jade C | interaction | F (1, 16) = 6.111 | 0.0250 | 0.276 |
|  | ouabain | F (1, 16) = 7.314 | 0.0156 | 0.314 |
|  | β-caryophyllene | F (1, 16) = 3.305 | 0.0878 | 0.171 |
| Dentate Gyrus Fluoro-Jade C | interaction | F (1, 16) = 7.528 | 0.0144 | 0.320 |
|  | ouabain | F (1, 16) = 9.738 | 0.0066 | 0.378 |
|  | β-caryophyllene | F (1, 16) = 4.252 | 0.0558 | 0.210 |

| **Table S3. Tukey comparisons and Cohen's d** | | | |
| --- | --- | --- | --- |
| **Variable** | **Comparison** | **p value** | **cohen's d** |
| Crossing | aCSF:Saline vs. aCSF:BCP | 0.9999 | 0.0397 |
|  | aCSF:Saline vs. OUA:Saline | 0.0108 | 1.7335 |
|  | aCSF:Saline vs. OUA:BCP | 0.9989 | 0.0782 |
|  | aCSF:BCP vs. OUA:Saline | 0.0127 | 2.1179 |
|  | aCSF:BCP vs. OUA:BCP | 0.9999 | 0.0521 |
|  | OUA:Saline vs. OUA:BCP | 0.0149 | 2.0366 |
| Distance traveled | aCSF:Saline vs. aCSF:BCP | 0.0837 | 1.3166 |
|  | aCSF:Saline vs. OUA:Saline | <0.0001 | 3.7068 |
|  | aCSF:Saline vs. OUA:BCP | 0.2167 | 1.1423 |
|  | aCSF:BCP vs. OUA:Saline | 0.0011 | 2.6548 |
|  | aCSF:BCP vs. OUA:BCP | 0.001 | 2.9543 |
|  | OUA:Saline vs. OUA:BCP | <0.0001 | 6.0100 |
| Time spent in central area | aCSF:Saline vs. aCSF:BCP | 0.4889 | 1.5132 |
|  | aCSF:Saline vs. OUA:Saline | 0.0482 | 1.3643 |
|  | aCSF:Saline vs. OUA:BCP | 0.8732 | 0.5772 |
|  | aCSF:BCP vs. OUA:Saline | 0.0020 | 2.0564 |
|  | aCSF:BCP vs. OUA:BCP | 0.9009 | 0.5211 |
|  | OUA:Saline vs. OUA:BCP | 0.0095 | 1.5833 |
| Visit to central area | aCSF:Saline vs. aCSF:BCP | 0.908 | 0.4675 |
|  | aCSF:Saline vs. OUA:Saline | 0.0326 | 1.8257 |
|  | aCSF:Saline vs. OUA:BCP | 0.958 | 0.2817 |
|  | aCSF:BCP vs. OUA:Saline | 0.1231 | 1.3860 |
|  | aCSF:BCP vs. OUA:BCP | 0.653 | 0.6440 |
|  | OUA:Saline vs. OUA:BCP | 0.011 | 1.7610 |
| Time in open arms | aCSF:Saline vs. aCSF:BCP | 0.2914 | 1.4152 |
|  | aCSF:Saline vs. OUA:Saline | 0.8732 | 0.4405 |
|  | aCSF:Saline vs. OUA:BCP | 0.9199 | 0.3833 |
|  | aCSF:BCP vs. OUA:Saline | 0.7116 | 0.5917 |
|  | aCSF:BCP vs. OUA:BCP | 0.6394 | 0.6811 |
|  | OUA:Saline vs. OUA:BCP | 0.9993 | 0.0584 |
| Closed arm entries | aCSF:Saline vs. aCSF:BCP | 0.437 | 1.0742 |
|  | aCSF:Saline vs. OUA:Saline | 0.0005 | 3.3697 |
|  | aCSF:Saline vs. OUA:BCP | 0.0009 | 2.4772 |
|  | aCSF:BCP vs. OUA:Saline | 0.0164 | 2.0820 |
|  | aCSF:BCP vs. OUA:BCP | 0.0285 | 1.5492 |
|  | OUA:Saline vs. OUA:BCP | 0.9939 | 0.1285 |
| Pick-up score | aCSF:Saline vs. aCSF:BCP | >0.9999 | 0 |
|  | aCSF:Saline vs. OUA:Saline | 0.0128 | 1.9026 |
|  | aCSF:Saline vs. OUA:BCP | 0.9332 | 0.3581 |
|  | aCSF:BCP vs. OUA:Saline | 0.0128 | 1.9026 |
|  | aCSF:BCP vs. OUA:BCP | 0.9332 | 0.3581 |
|  | OUA:Saline vs. OUA:BCP | 0.0456 | 1.4411 |
|  |  |  |  |
|  |  |  |  |
| **Table S3. Tukey comparisons and Cohen's d (continued)** | | | |
| **Variable** | **Comparison** | **p value** | **cohen's d** |
| GST | aCSF:Saline vs. aCSF:BCP | 0.9986 | 0.0770 |
|  | aCSF:Saline vs. OUA:Saline | 0.0413 | 1.8344 |
|  | aCSF:Saline vs. OUA:BCP | 0.7548 | 0.6678 |
|  | aCSF:BCP vs. OUA:Saline | 0.0567 | 1.4064 |
|  | aCSF:BCP vs. OUA:BCP | 0.8357 | 0.4471 |
|  | OUA:Saline vs. OUA:BCP | 0.2601 | 1.3674 |
| SOD | aCSF:Saline vs. aCSF:BCP | 0.9534 | 0.3252 |
|  | aCSF:Saline vs. OUA:Saline | 0.0006 | 3.2584 |
|  | aCSF:Saline vs. OUA:BCP | 0.5968 | 0.6577 |
|  | aCSF:BCP vs. OUA:Saline | 0.002 | 2.8006 |
|  | aCSF:BCP vs. OUA:BCP | 0.3111 | 0.9086 |
|  | OUA:Saline vs. OUA:BCP | <0.0001 | 3.2581 |
| CAT | aCSF:Saline vs. aCSF:BCP | 0.9907 | 0.1816 |
|  | aCSF:Saline vs. OUA:Saline | 0.0356 | 1.8554 |
|  | aCSF:Saline vs. OUA:BCP | 0.9237 | 0.4824 |
|  | aCSF:BCP vs. OUA:Saline | 0.0189 | 1.5653 |
|  | aCSF:BCP vs. OUA:BCP | 0.9876 | 0.1758 |
|  | OUA:Saline vs. OUA:BCP | 0.0092 | 1.9602 |
| TBARs | aCSF:Saline vs. aCSF:BCP | 0.9899 | 0.2254 |
|  | aCSF:Saline vs. OUA:Saline | 0.003 | 2.2077 |
|  | aCSF:Saline vs. OUA:BCP | 0.3167 | 1.1699 |
|  | aCSF:BCP vs. OUA:Saline | 0.006 | 1.9582 |
|  | aCSF:BCP vs. OUA:BCP | 0.1959 | 1.2886 |
|  | OUA:Saline vs. OUA:BCP | <0.0001 | 2.8605 |
| NRF2 | aCSF:Saline vs. aCSF:BCP | 0.8903 | 0.3972 |
|  | aCSF:Saline vs. OUA:Saline | 0.0078 | 2.2178 |
|  | aCSF:Saline vs. OUA:BCP | 0.9138 | 0.3439 |
|  | aCSF:BCP vs. OUA:Saline | 0.0371 | 1.9011 |
|  | aCSF:BCP vs. OUA:BCP | >0.9999 | 0.0345 |
|  | OUA:Saline vs. OUA:BCP | 0.0325 | 1.8070 |
| total GSK-3β | aCSF:Saline vs. aCSF:BCP | 0.9247 | 0.3696 |
|  | aCSF:Saline vs. OUA:Saline | 0.119 | 1.1673 |
|  | aCSF:Saline vs. OUA:BCP | 0.6772 | 0.7838 |
|  | aCSF:BCP vs. OUA:Saline | 0.3325 | 0.8754 |
|  | aCSF:BCP vs. OUA:BCP | 0.9561 | 0.3659 |
|  | OUA:Saline vs. OUA:BCP | 0.6176 | 0.6869 |
| phospho GSK-3β | aCSF:Saline vs. aCSF:BCP | >0.9999 | 0.0232 |
|  | aCSF:Saline vs. OUA:Saline | 0.2041 | 1.2250 |
|  | aCSF:Saline vs. OUA:BCP | 0.4358 | 1.2998 |
|  | aCSF:BCP vs. OUA:Saline | 0.1902 | 0.9743 |
|  | aCSF:BCP vs. OUA:BCP | 0.4593 | 0.8348 |
|  | OUA:Saline vs. OUA:BCP | 0.0092 | 2.1640 |
|  |  |  |  |
|  |  |  |  |
| **Table S3. Tukey comparisons and Cohen's d (continued)** | | | |
| **Variable** | **Comparison** | **p value** | **cohen's d** |
| GSK-3β ratio | aCSF:Saline vs. aCSF:BCP | 0.2838 | 0.8425 |
|  | aCSF:Saline vs. OUA:Saline | <0.0001 | 2.8900 |
|  | aCSF:Saline vs. OUA:BCP | 0.8091 | 0.4261 |
|  | aCSF:BCP vs. OUA:Saline | 0.0008 | 3.6649 |
|  | aCSF:BCP vs. OUA:BCP | 0.7783 | 0.9881 |
|  | OUA:Saline vs. OUA:BCP | <0.0001 | 5.0903 |
| total PKA | aCSF:Saline vs. aCSF:BCP | 0.0942 | 1.5414 |
|  | aCSF:Saline vs. OUA:Saline | 0.3144 | 0.9759 |
|  | aCSF:Saline vs. OUA:BCP | 0.7455 | 0.5877 |
|  | aCSF:BCP vs. OUA:Saline | 0.8937 | 0.4042 |
|  | aCSF:BCP vs. OUA:BCP | 0.4735 | 0.8946 |
|  | OUA:Saline vs. OUA:BCP | 0.8697 | 0.4135 |
| phospho PKA | aCSF:Saline vs. aCSF:BCP | 0.9675 | 0.2484 |
|  | aCSF:Saline vs. OUA:Saline | 0.0083 | 2.1671 |
|  | aCSF:Saline vs. OUA:BCP | 0.6476 | 0.8421 |
|  | aCSF:BCP vs. OUA:Saline | 0.003 | 2.0321 |
|  | aCSF:BCP vs. OUA:BCP | 0.383 | 0.9146 |
|  | OUA:Saline vs. OUA:BCP | 0.099 | 1.5173 |
| PKA ratio | aCSF:Saline vs. aCSF:BCP | <0.0001 | 7.0206 |
|  | aCSF:Saline vs. OUA:Saline | <0.0001 | 3.3338 |
|  | aCSF:Saline vs. OUA:BCP | 0.979 | 0.2076 |
|  | aCSF:BCP vs. OUA:Saline | <0.0001 | 10.582 |
|  | aCSF:BCP vs. OUA:BCP | <0.0001 | 6.7181 |
|  | OUA:Saline vs. OUA:BCP | <0.0001 | 2.9621 |
| CA1 Fluoro-Jade C | aCSF:Saline vs. aCSF:BCP | 0.9949 | 0.3439 |
|  | aCSF:Saline vs. OUA:Saline | 0.0012 | 2.1682 |
|  | aCSF:Saline vs. OUA:BCP | 0.9856 | 0.4198 |
|  | aCSF:BCP vs. OUA:Saline | 0.0007 | 2.3801 |
|  | aCSF:BCP vs. OUA:BCP | 0.9996 | 0.1934 |
|  | OUA:Saline vs. OUA:BCP | 0.0006 | 2.3812 |
| CA3 Fluoro-Jade C | aCSF:Saline vs. aCSF:BCP | 0.9662 | 0.4592 |
|  | aCSF:Saline vs. OUA:Saline | 0.0102 | 1.9079 |
|  | aCSF:Saline vs. OUA:BCP | 0.9219 | 0.7843 |
|  | aCSF:BCP vs. OUA:Saline | 0.0259 | 1.5312 |
|  | aCSF:BCP vs. OUA:BCP | 0.9983 | 0.1432 |
|  | OUA:Saline vs. OUA:BCP | 0.0358 | 1.5192 |
| Dentate Gyrus Fluoro-Jade C | aCSF:Saline vs. aCSF:BCP | 0.962 | 0.8522 |
|  | aCSF:Saline vs. OUA:Saline | 0.0038 | 2.0334 |
|  | aCSF:Saline vs. OUA:BCP | 0.876 | 0.9513 |
|  | aCSF:BCP vs. OUA:Saline | 0.0101 | 1.7509 |
|  | aCSF:BCP vs. OUA:BCP | 0.9931 | 0.2907 |
|  | OUA:Saline vs. OUA:BCP | 0.0174 | 1.5709 |

| **Supplementary Table S4. Group descriptive statistics** | | | | | | | | | | | | |
| --- | --- | --- | --- | --- | --- | --- | --- | --- | --- | --- | --- | --- |
| **Variable** | **Saline + aCSF** | | | **BCP + aCSF** | | | **Saline + OUA** | | | **BCP + OUA** | | |
|  | **Mean** | **Standart deviation** | | **Mean** | **Standart deviation** | | **Mean** | **Standart deviation** | | **Mean** | **Standart deviation** | |
| Crossing | 102.0 | 15.2 | | 102.5 | 9.4 | | 126.5 | 13.0 | | 103.0 | 9.8 | |
| Distance traveled | 16.5 | 3.0 | | 20.1 | 2.4 | | 26.4 | 2.3 | | 13.7 | 1.9 | |
| Time spent in central area | 7.0 | 2.3 | | 3.5 | 2.3 | | 13.8 | 6.7 | | 5.2 | 3.9 | |
| Visit to central area | 5.5 | 1.4 | | 6.2 | 1.5 | | 8.5 | 1.9 | | 5.0 | 2.1 | |
| Time in open arms | 139.3 | 30.1 | | 119.7 | 55.5 | | 91.8 | 36.7 | | 122.8 | 52.9 | |
| Closed arm entries | 8.7 | 0.8 | | 7.7 | 1.0 | | 5.5 | 1.0 | | 5.7 | 1.5 | |
| GST | 145.8 | 18.7 | | 144.0 | 26.0 | | 113.3 | 16.7 | | 134.6 | 14.5 | |
| SOD | 21.1 | 3.0 | | 22.1 | 3.2 | | 30.3 | 2.7 | | 18.7 | 4.3 | |
| CAT | 3.5 | 0.6 | | 3.3 | 1.3 | | 5.4 | 1.3 | | 3.1 | 1.0 | |
| TBARs | 67.2 | 14.3 | | 70.7 | 16.9 | | 114.5 | 26.7 | | 46.7 | 20.3 | |
| NRF2 | 100.0 | ‘12.4 | | 95.3 | 11.2 | | 76.0 | 9.0 | | 95.7 | 12.5 | |
| total GSK-3β | 100.0 | 30.7 | | 111.1 | 29.2 | | 142.2 | 40.8 | | 120.2 | 19.5 | |
| phospho GSK-3β | 100.0 | 13.6 | | 100.5 | 25.3 | | 76.9 | 22.9 | | 117.3 | 13.0 | |
| GSK-3β ratio | 1.6 | 0.3 | | 1.4 | 0.1 | | 0.8 | 0.2 | | 1.5 | 0.1 | |
| total PKA | 100.0 | 15.6 | | 77.1 | 14.1 | | 83.6 | 17.9 | | 90.7 | 16.2 | |
| phospho PKA | 100.0 | 13.5 | | 104.2 | 19.8 | | 66.6 | 17.1 | | 89.2 | 12.2 | |
| PKA ratio | 0.7 | 0.0 | | 1.0 | 0.0 | | 0.6 | 0.0 | | 0.7 | 0.0 | |
| CA1 Fluoro-Jade C | 1.4 | 1.2 | | 1.1 | 0.4 | | 7.6 | 3.8 | | 1.0 | 0.9 | |
| CA3 Fluoro-Jade C | 1.4 | 1.1 | | 2.2 | 2.4 | | 8.1 | 4.8 | | 2.5 | 1.8 | |
| Dentate Gyrus Fluoro-Jade C | 0.5 | 0.6 | | 1.5 | 1.4 | | 8.6 | 5.6 | | 2.0 | 2.1 | |
| **Variable** | **Saline + aCSF** | | | **BCP + aCSF** | | | **Saline + OUA** | | | **BCP + OUA** | | |
|  | **Median** | **Upper Limit** | **Lower Limit** | **Median** | **Upper Limit** | **Lower Limit** | **Median** | **Upper Limit** | **Lower Limit** | **Median** | **Upper Limit** | **Lower Limit** |
| Pick-up score | 1 | 1.25 | 1 | 1 | 1.25 | 1 | 2.5 | 4.25 | 1.75 | 1 | 2 | 1 |

**‘**

**Western blot membranes of NRF2 (Fig. 4E), GSK-3β (Fig. 5A), phospho-GSK-3β (Fig. 5B), PKA (Fig. 5D), and phospho-PKA (Fig. 5E).** Due to the number of samples and gel capacity limitations, two independent membranes were generated for each target protein (M1 and M2). Treatment conditions for each sample are indicated above the respective lanes using (+) or (−) symbols, representing the presence or absence of ouabain (OUA) and beta-caryophyllene (BCP), respectively. Densitometric analyses were performed using the original full-length, uncropped membranes prior to any image processing. Background areas unrelated to the membranes were removed for presentation purposes.

**
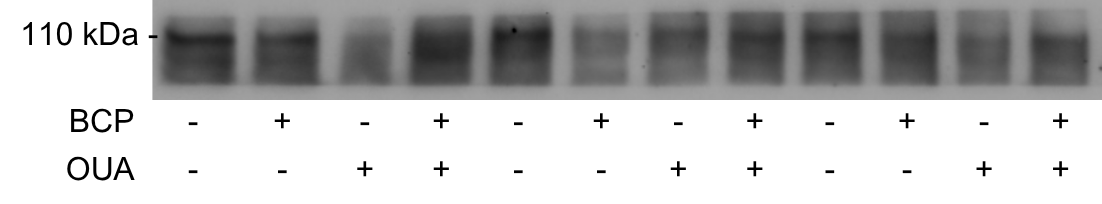
NRF2 – M1**

**
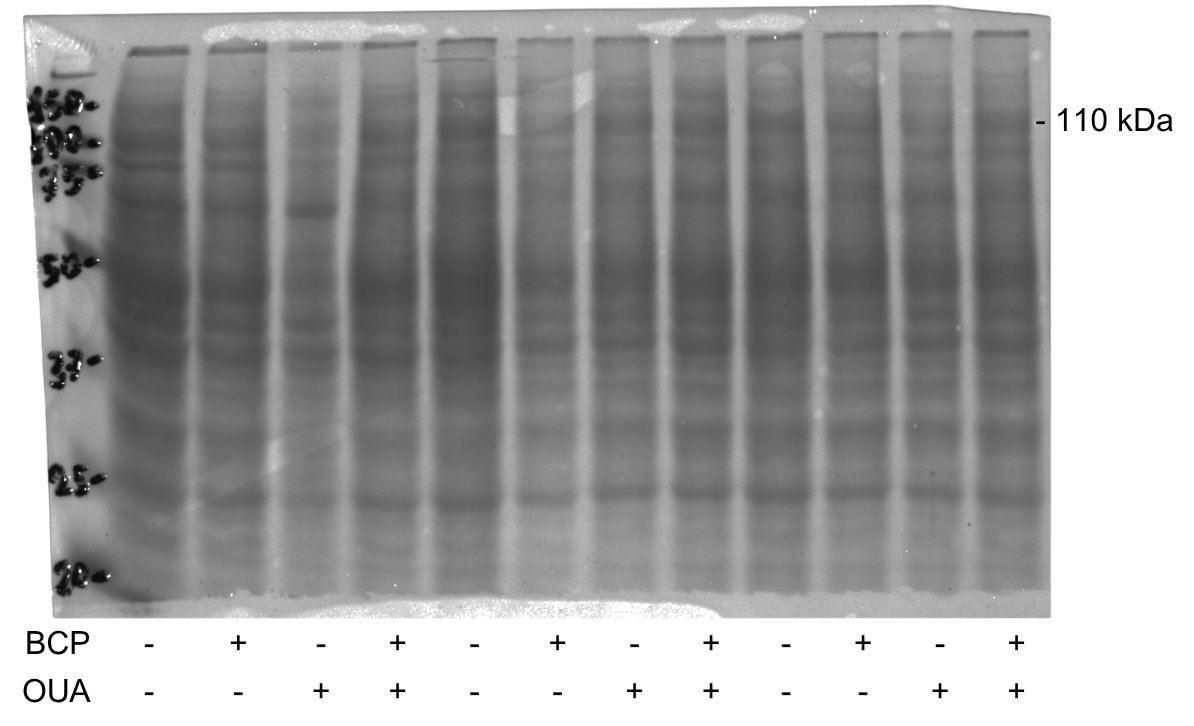
NRF2 – M1 – PonceauS**

**
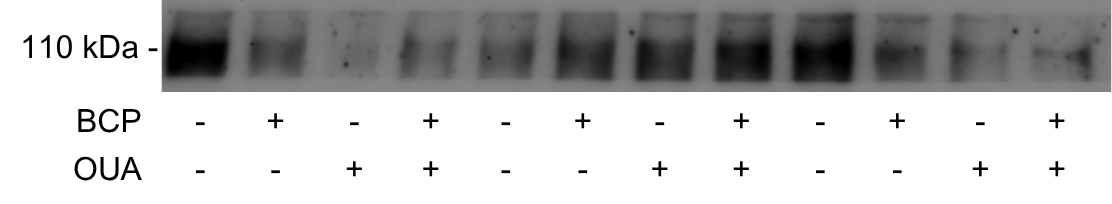
NRF2 – M2**

**
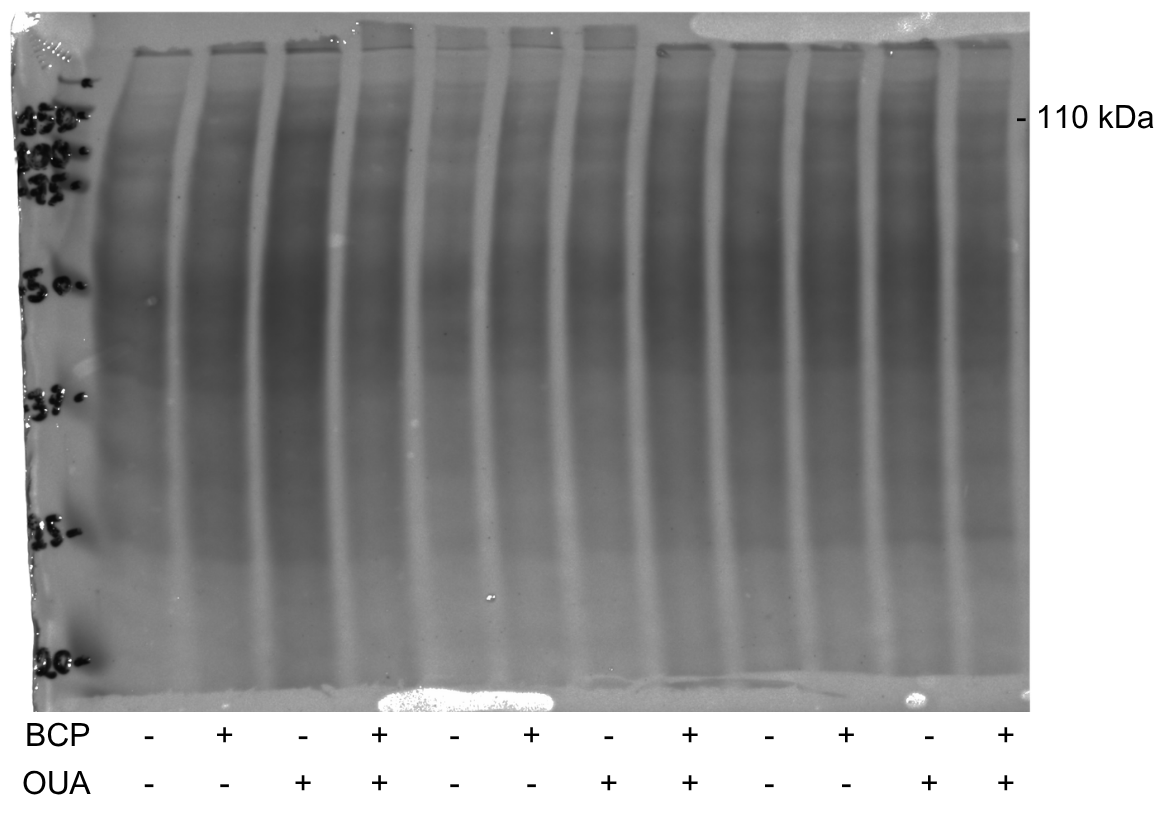
NRF2 – M2 – PonceauS**

**
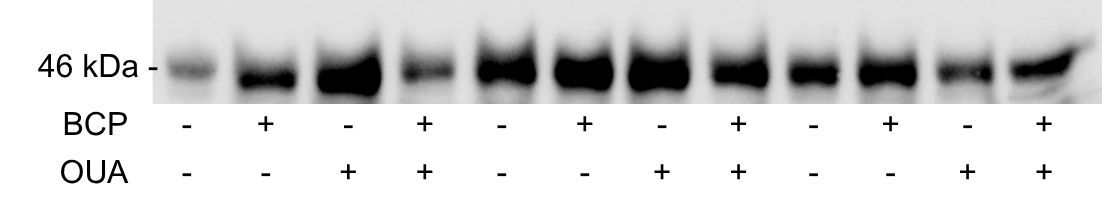
GSK-3β – M1**

**
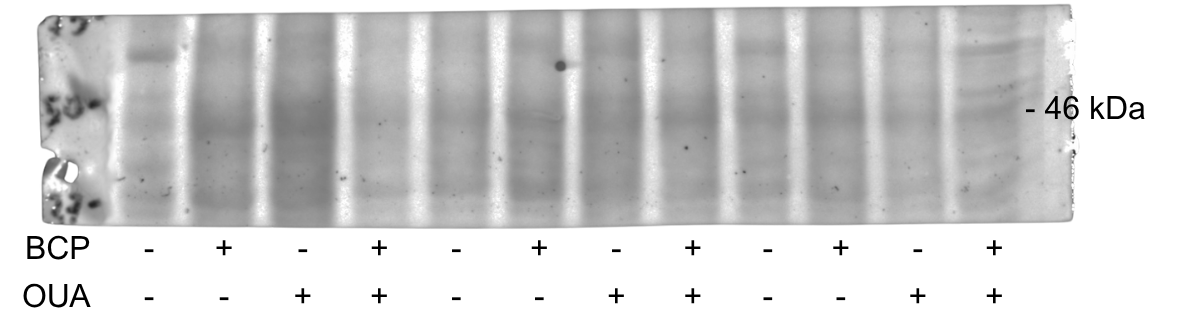
GSK-3β – M1 – PonceauS**

**
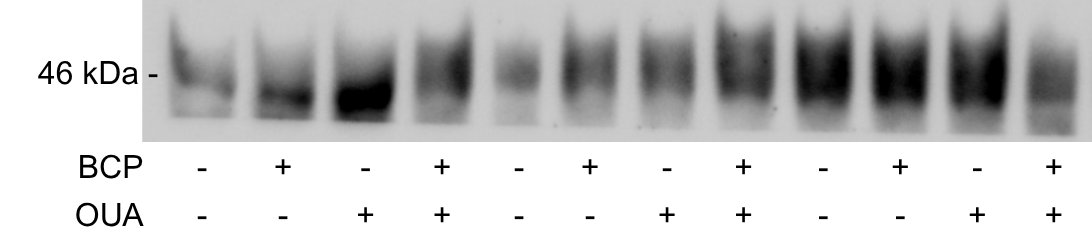
GSK-3β – M2**

**
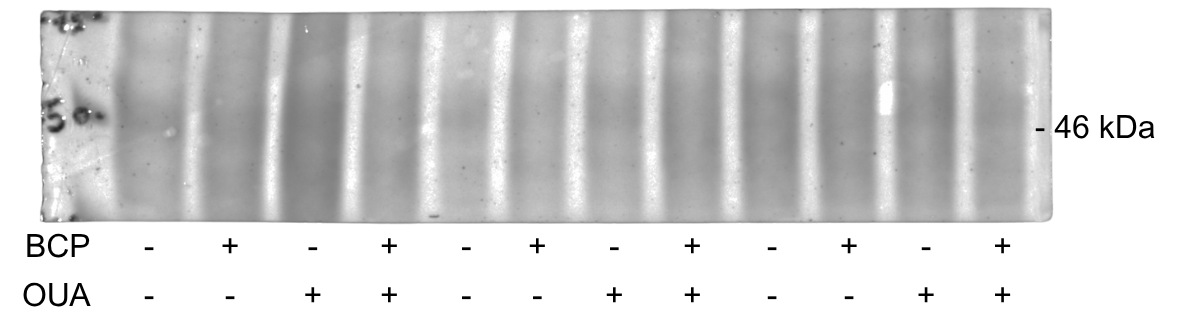
GSK-3β – M2 – PonceauS**

**
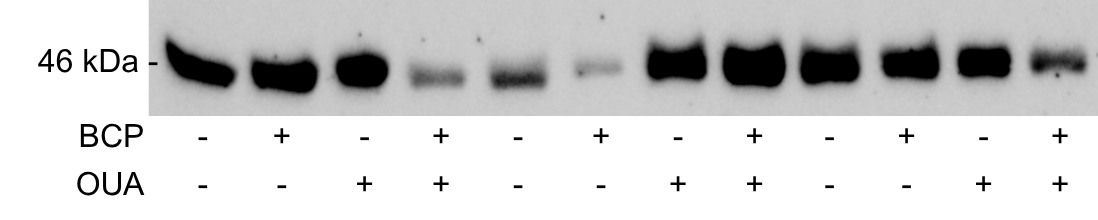
Phospho-GSK-3β (Ser^9^) – M1**

**
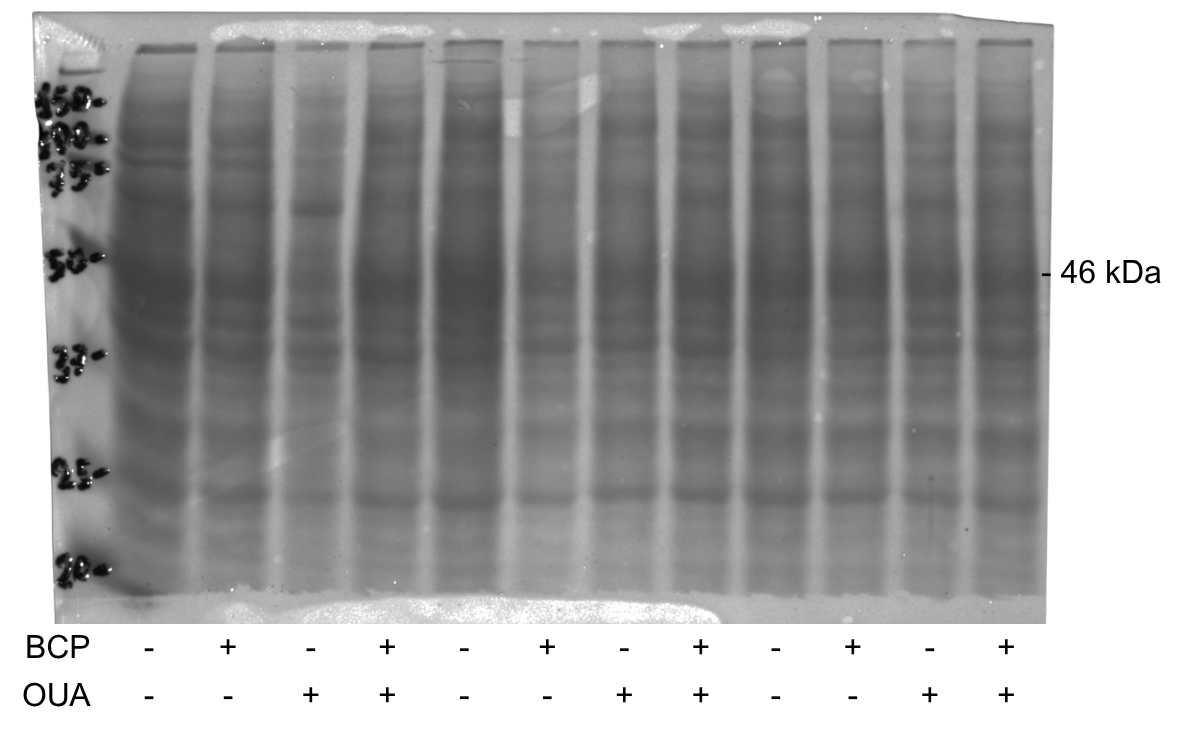
Phospho-GSK-3β (Ser^9^) – M1 – PonceauS**

**
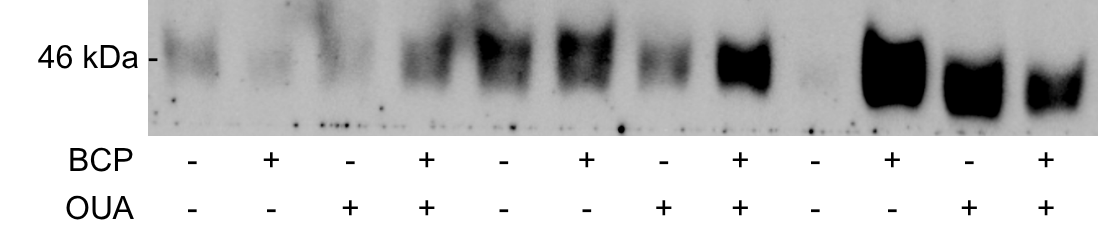
Phospho-GSK-3β (Ser^9^) – M2**

**
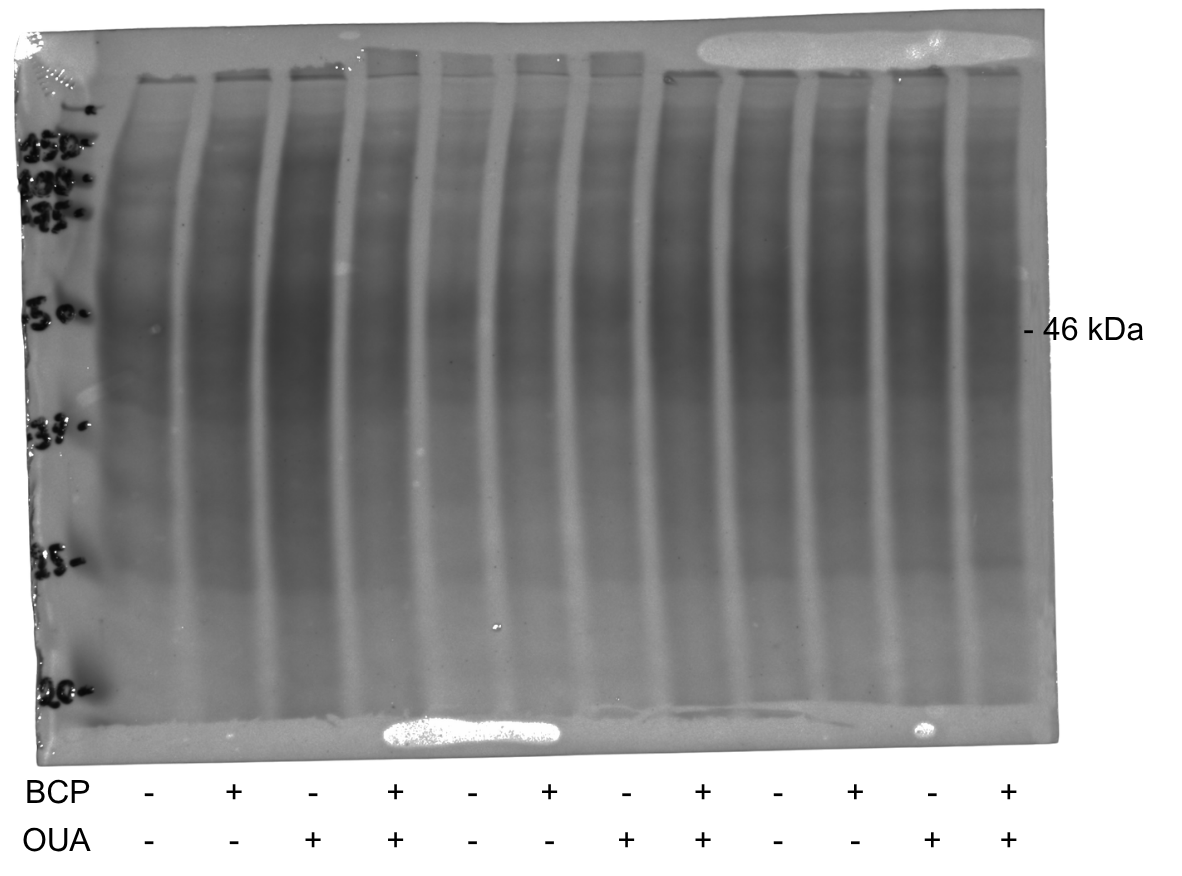
Phospho-GSK-3β (Ser^9^) – M2 – PonceauS**

**
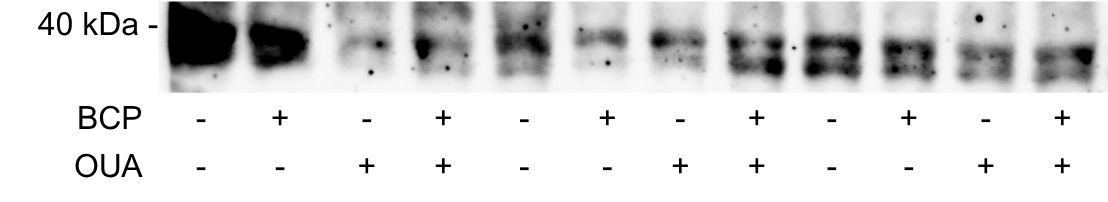
PKA – M1**

**
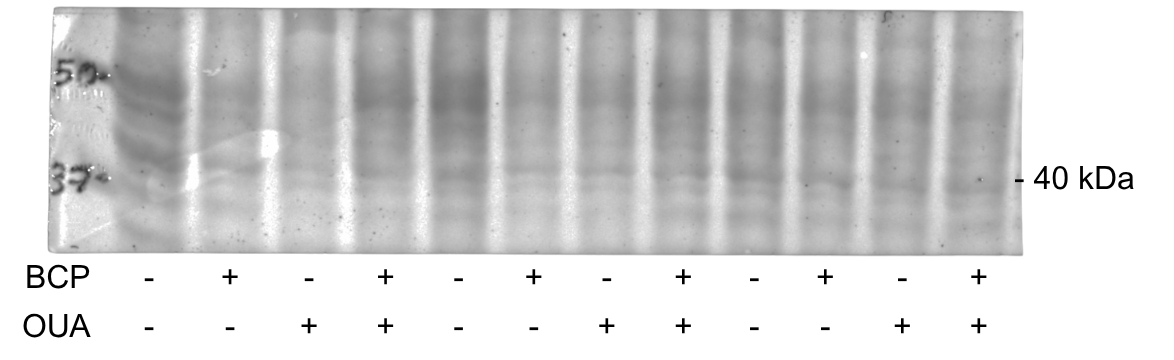
PKA – M1 – PonceauS**

**
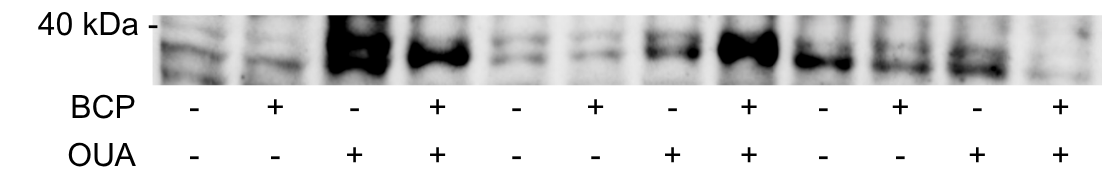
PKA – M2**

**
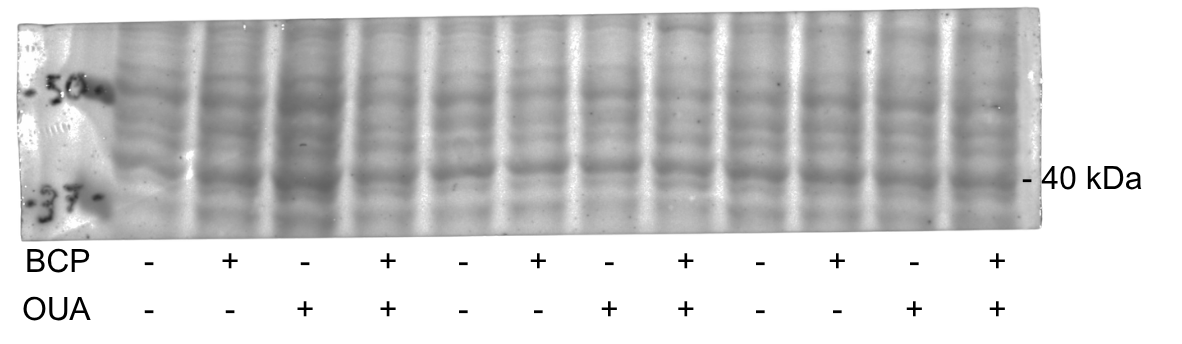
PKA – M2 – PonceauS**

**
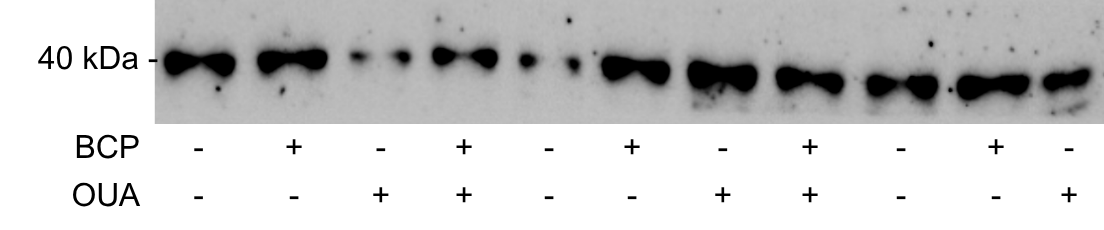
Phospho-PKA (Ser^96^) – M1**

**
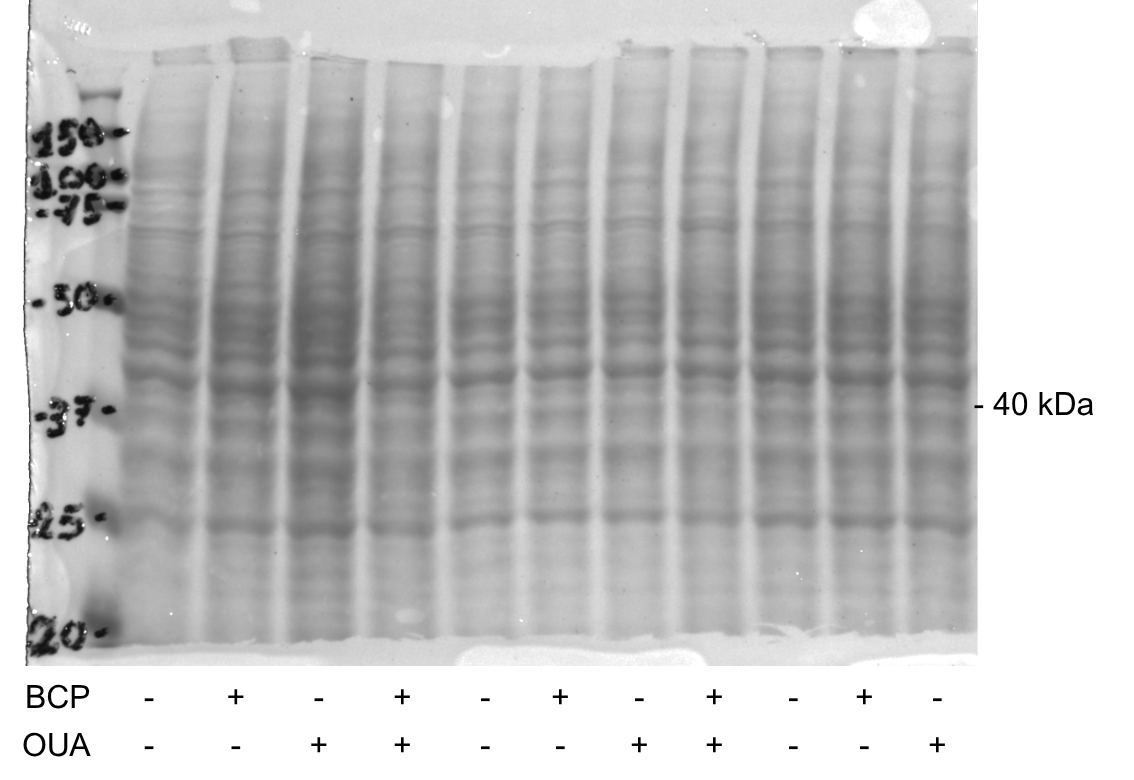
Phospho-PKA (Ser^96^) – M1 – PonceauS**

**
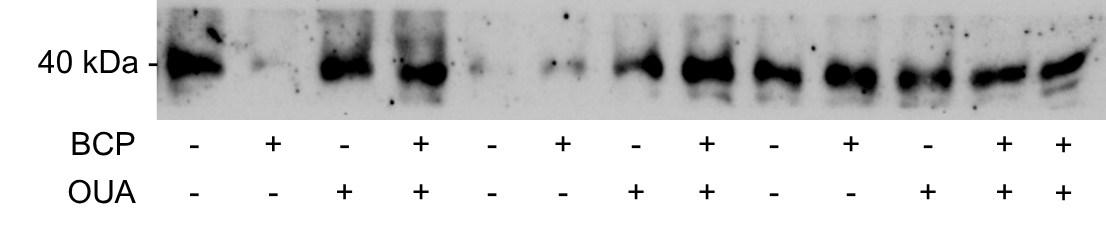
Phospho-PKA (Ser^96^) – M2**

**
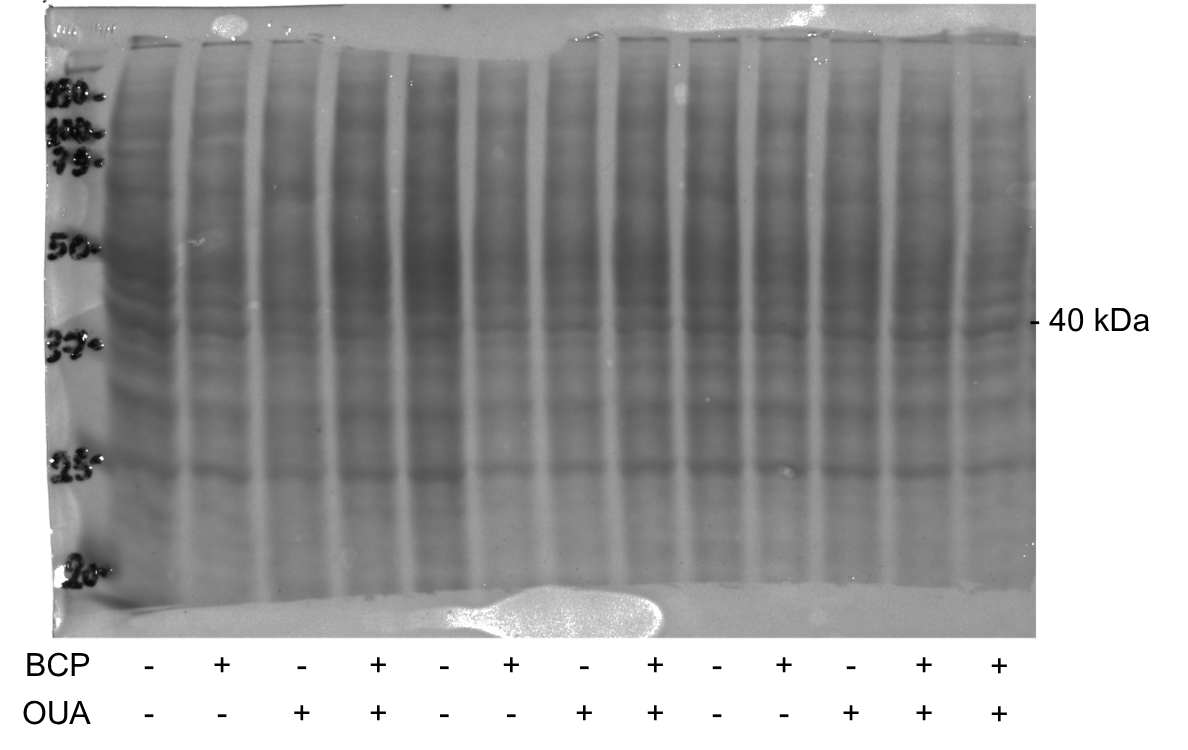
Phospho-PKA (Ser^96^) – M2 – PonceauS**
